# Supplementary material for: Early-life adversity alters adult nucleus incertus neurons: implications for neuronal mechanisms of increased stress and compulsive behavior vulnerability
Source: Neuropsychopharmacology. 2025 Mar 21;50(9):1406–19. doi: 10.1038/s41386-025-02089-0 (PMC12259866; doi:10.1038/s41386-025-02089-0)
Supplement: Supplementary file 1 — Supplementary Materials [file 41386_2025_2089_MOESM1_ESM.pdf]

Neuropsychopharmacology NPP-24-1366R1

Supplementary Materials

Early-life adversity alters adult nucleus incertus neurons: implications for neuronal mechanisms of increased stress and compulsive behavior vulnerability

Anna Gugula[1]*, Patryk Sambak[1,4#], Aleksandra Trenk[1#], Sylwia Drabik[1,4], Aleksandra Nogaj[1,4], Zbigniew Soltys[2], Andrew L. Gundlach[3], and Anna Blasiak[1]*

[1]*Department of Neurophysiology and Chronobiology, Institute of Zoology and Biomedical Research, Faculty of Biology, Jagiellonian University, Krakow, Poland*

[2]*Laboratory of Experimental Neuropathology, Institute of Zoology and Biomedical Research, Faculty of Biology, Jagiellonian University, Krakow, Poland*

[3]*The Florey Institute of Neuroscience and Mental Health, and Florey Department of Neuroscience and Mental Health, and Department of Anatomy and Physiology, The University of Melbourne, Parkville, Victoria, Australia*

[4]*Doctoral School of Exact and Natural Sciences, Jagiellonian University, Krakow, Poland*

Materials and Methods

Animals and treatment

Male Sprague-Dawley rats (control, Ctrl, n = 33, from 8 litters; and subjected to a maternal separation procedure, MS, n = 39, from 9 litters) from the Institute of Zoology and Biomedical Research Animal Facility of the Jagiellonian University in Krakow, Poland, were used in the described experiments. The rats were housed with food and water available *ad libitum*, on a standard light/dark cycle (lights on 08.00–20.00), temperature 22-23°C. The experiments were approved by the Second Local Institutional Animal Care and Use Committee (Krakow, Poland) and conducted in accordance with the EU Directive 2010/63/EU on the protection of animals used for scientific purposes. All efforts were made to minimize the number of rats used and their suffering.

Maternal separation procedure

Pregnant dams, housed as described, were selected and isolated from males to avoid reimpregnation and bearing a second litter during the MS procedure. Litters were standardized to a fixed number of 5–9 male pups. The day of birth was defined as postnatal day 0 (PND 0), and the MS procedure was conducted during PND 2–14, as described (1). Briefly, the dams were moved to separate holding cages for 3 h daily (Zeitgeber time (ZT) 3–6), leaving the litters alone as a group in their home cage. On PND 28 the rats were weaned, transferred to separate cages, and housed as family groups until use in further experimental procedures.

Acute stress and c-Fos expression in neurochemically-defined nucleus incertus neurons

Restraint stress procedure and brain fixation

On PND 55–56, Ctrl and MS rats were subjected to 30 min restraint stress in the experimental room (control stressed, Ctrl S, n = 5; MS stressed, MS S, n = 6), to determine the influence of MS on stress-induced c-Fos protein expression in the NI. Briefly, rats were restrained for 30 min in custom-made disposable transparent plastic bag restrainers with a breathing hole at the tip, during ZT 1–5. As a control condition, rats from Ctrl (n = 6, Ctrl NS) and MS (n = 5, MS NS) groups were kept in transport cages in the experimental room for 30 min without restraint (rats from the other groups were restrained on a different day). At 1 h (sufficient time for c-Fos protein expression; (2)) after the end of the restraint procedure (Ctrl S and MS S groups) or after the end of the exposure to the experimental room (Ctrl NS and MS NS groups), rats from all groups were lightly anesthetized with isoflurane (Aerrane, Baxter, Poland) and deeply anesthetized with pentobarbital (Morbital, Biowet, Poland; intraperitoneally, dose: 2 ml/kg). Subsequently, rats were transcardially perfused with phosphate-buffered saline (PBS, 4°C, pH 7.4, ~250 ml) followed by 4% phosphate-buffered formaldehyde solution (4°C, pH 7.4, ~250 ml). At the end of the procedure, brains were collected and postfixed overnight (4% formaldehyde, 4°C).

Immunohistochemical staining, imaging and cell counting

After post-fixation, brains were washed in PBS and transferred to 30% sucrose solution in PBS (4°C, ~24 h). Next, the brainstem area containing the NI was cut into 45 µm coronal sections on a freezing microtome. Every fourth section was incubated in 10% normal donkey serum (NDS, Jackson ImmunoResearch, West Grove, PA, USA) and 0.3% Triton X-100 (Sigma-Aldrich, cat. no. X100) PBS solution (1 h, RT). Sections were then transferred into a

solution containing primary antibodies against relaxin-3 (RLN3, mouse monoclonal, 1:10, provided by The Florey Institute of Neuroscience and Mental Health, Parkville, Australia) and c-Fos (rabbit polyclonal, 1:10000, Abcam, Cambridge, UK, cat. no. ab190289), 2% NDS and 0.3% Triton X-100 in PBS, and incubated for ~72 h at 4°C. After washing in PBS, sections were placed in a secondary antibody solution, containing Alexa Fluor 488 anti-mouse (donkey, 1:400, Jackson ImmunoResearch, cat. no. 715-546-150), Cy3 anti-rabbit (donkey, 1:400, Jackson ImmunoResearch, cat. no. 711-165-152) and 2% NSD in PBS at 4°C, overnight. Sections were then washed, and all described steps were repeated, with a primary antibody against pro-cholecystokinin (pCCK, rabbit polyclonal, 1:200, Frontier Institute Co., Ltd, Hokkaido, Japan, cat. no. CCK-pro-RB-Af350). After washing in PBS, sections were incubated with Alexa Fluor 647 anti-rabbit secondary antibody (donkey, 1:400, Jackson ImmunoResearch, cat. no. 711-606-152; overnight, 4°C) and mounted on glass slides using Fluoroshield with DAPI (Sigma Aldrich, cat# F6057).

Z-stack panoramic images of matching brainstem sections containing the NI (three per brain) were collected using an Axio Imager M2 fluorescence microscope (Zeiss, Gottingen, Germany), equipped with an automatic z-stage, Axiocam 503 mono camera (Zeiss) EC Plan-Neofluar 20×/0.50 M27 objective (scaling: 0.227 × 0.227 × 1.380 µm/pixel, respectively in the x, y and z axis). To improve the signal-to-noise ratio, the images were subsequently postprocessed with Zen 3.1 blue edition (Zeiss) and ImageJ software (3).

Neurons immunopositive for RLN3 (RLN3+) and pro-CCK (pCCK+), co-expressing c-Fos protein (c-Fos+) or without such co-expression (c-Fos–), were counted using an ImageJ Cell Counter plugin. Since the rat NI has been anatomically divided into the pars compacta (NIc) and pars dissipata (NId) (4), cells in the NIc and NId were counted separately. Neurons immunopositive for both RLN3 and pCCK were excluded from the analysis due to the rarity of their occurrence.

Electrophysiological patch clamp recordings

A total of 38 male rats (5–8 weeks old, Ctrl group; n = 17, MS group; n = 21) were used in electrophysiological experiments. Rats were deeply anesthetized with isoflurane (Aerrane, Baxter, Poland) and decapitated (ZT 1–2). Brains were dissected from the skull into carbogenated, ice-cold artificial cerebrospinal fluid (ACSF), containing (in mM): 185 sucrose, 25 $NaHCO_3$, 3 KCl, 1.2 NaH_2PO_4, 2 $CaCl_2$, 10 $MgSO_4$ and 10 glucose, pH 7.4; osmolality 290–300 mOsmol/kg) and cut into 250 µm coronal slices on a Leica VT 1000S vibrating microtome (Leica Instruments, Heidelberg, Germany). NI containing slices were subsequently transferred to an incubation chamber containing warm (initially at 32°C), carbogenated standard ACSF (sACSF) containing (in mM): 118 NaCl, 25 $NaHCO_3$, 3 KCl, 1.2 NaH_2PO_4, 2 $CaCl_2$, 2 $MgSO_4$, and 10 glucose (pH 7.4; osmolality 290–300 mOsmol/kg).

After at least 90 min of recovery, slices were transferred to the recording chamber, with constant perfusion (2 ml/min) of fresh, warm (32°C), carbogenated sACSF. The recording chamber was mounted on the fixed stage of an Axioscope 2 microscope (Zeiss). Whole-cell configuration was obtained under visual control with negative pressure obtained by pressure controller (ez-gSEAL 100B, NeoBiosytems, San José, CA, USA), delivered through recordings micropipettes of resistance between 5 and 8 MΩ. Recording micropipettes were pulled on a horizontal puller (P-1000, Sutter Instruments, Novato, CA, USA) using borosilicate glass capillaries (Sutter Instruments). The pipette solution contained (in mM):

125 potassium gluconate, 2 $MgCl_2$, 4 Na_2ATP, 0.4 Na_3GTP, 5 EGTA, 10 HEPES (pH 7.3; osmolality 290–300 mOsmol/kg) and biocytin (0.05%) which allowed for subsequent immunofluorescent identification of recorded neurons. The calculated liquid junction potential for sACSF and the intrapipette solution was +15 mV, and this value was subtracted from the data. All reagents used for the ACSF and intrapipette solution were purchased from Sigma-Aldrich (Darmstadt, Germany). An SEC 05LX amplifier (NPI, Tamm, Germany) and Signal and Spike2 (Cambridge Electronic Design, Cambridge, UK) software were used for data acquisition and further analysis (low-pass filtered at 3 kHz and digitized at 20 kHz), as well as a custom-made script in MATLAB (MathWorks Inc., Natick, MA, USA). All drugs were delivered via a bath perfusion system.

The experimental protocols used in voltage-clamp conditions (holding voltage –75 mV) were as follows; (a) voltage ramp (1.3 s, –120 mV to +20 mV), (b) voltage steps (0.5 s, –120 mV to 0 mV, 10 mV increments), (c) test for potassium A current (hyperpolarization to –100 mV for 0.5 s and subsequent 0.5 s voltage steps from –75 mV to 25 mV, 10 mV increments); and in current-clamp conditions (membrane potential set at –75 mV); current ramp (1 s, 0 nA to 1 nA), current steps (0.5 s, –150 pA to +150 pA, 10 pA increments), single pulse to evoke action potential (2 ms, 0.75 nA). Next, the spontaneous activity of the recorded neurons was examined under current-clamp conditions (0 pA holding current) for at least 300 s, and subsequently spontaneous postsynaptic currents (sPSCs) were recorded under voltage-clamp conditions (holding potential –50 mV), for at least 300 s. Calculated reversal potential for Cl, Na and K currents in our patch-clamp recordings equaled –90.46, +72.33 and –98.02 mV respectively; therefore, at the –50mV holding potential, outward events represented IPSCs, whereas inward events represented EPSCs.

For examination of the miniature postsynaptic currents (mPSCs), a separate group of neurons was recorded in the presence of tetrodotoxin (TTX, 0.5 μM, Tocris Bioscience, Cat No. 1069, Bristol, UK) in ACSF. These neurons underwent the same tests in voltage- and current-clamp protocols. Both sPSCs and mPSCs were detected offline and analyzed using Mini Analysis software (Synaptosoft Inc., Fort Lee, NJ, USA) and were performed by experimenters blinded to the treatment conditions.

Post-recording immunostaining

After the electrophysiological recordings, brain slices were transferred to 4% formaldehyde in PBS (24 h, 4°C). Slices were washed in PBS and incubated in PBS solution containing 10% NDS, 0.6% Triton X-100 (overnight, 4°C). Next, after another washing in PBS, slices were incubated in PBS solution containing primary antibodies against RLN3 (1:25) and pCCK (1:200), ExtrAvidin-Cy3 (biocytin binding protein, 1:200, Sigma-Aldrich, cat. no. E4142), 2% NDS and 0.3% Triton X-100 for 48–72 h at 4°C. Subsequently, slices were washed in PBS and incubated in PBS solution containing 2% NDS, Alexa Fluor 647-conjugated donkey anti-rabbit (1:400, Jackson ImmunoResearch, cat. no. 711-605-152,) and Alexa Fluor 488-conjugated donkey anti-mouse secondary antibodies (1:400, 24 h, 4°C). After a final washing, slices were mounted on glass slides and coverslipped with Fluoroshield with DAPI.

Dendritic tracing and morphological analysis

After patch-clamp recordings, the influence of MS on the dendritic tree morphology of different types of NI neurons was examined in electrophysiological type I RLN3-immunopositive (Ctrl, n = 10, from 5 rats, and MS, n = 10, from 9 rats) and pro-CCK-

immunopositive (pCCK, Ctrl, n = 12, from 8 rats, and MS, n = 12, from 7 rats) neurons, and in electrophysiological type II cells (Ctrl, n = 10, from 6 rats, and MS, n = 13, from 6 rats) that were well-stained against biocytin, with clearly visible dendritic trees and with no evident truncations. Selected NI neurons were imaged using a confocal laser scanning microscope (LSM 780 on Axio Observer Z1, Zeiss) with a Plan-Apochromat 20×/0.8 M27 objective (scaling: 0.17 × 0.17 × 1.80 µm/pixel, respectively in the x, y and z axis). Subsequently, they were subjected to semi-automatic dendritic tracing using Simple Neurite Tracer plugin in ImageJ and saved as *.swc files for further analysis (5). Simultaneously a built-in function was used to conduct 3D Sholl analysis (6), with a 10 µm step size between virtual spheres intersecting with dendrites. The number of intersections per single radius, the sum of intersections and the maximum number of intersections per radius in each studied group of cells were analyzed. Finally, L-Measure software (7) was used to obtain topological parameters of the dendritic tree.

Multiplex fluorescent *in situ* hybridization

Multiplex fluorescent *in situ* hybridization (RNAscope™ HiPlex Assay for AF488, Atto550 and Atto647 detection, Advanced Cell Diagnostics (ACD), Hayward, CA, USA) was performed using 16 µm fresh-frozen brain sections from 5 Ctrl and 7 MS rats (PND 51–57; 3 matched NI containing slices/brain; brains collected during ZT 4–7). All procedures were performed following the manufacturer's user manual as described (8). The following probes were applied for round 1 (R1): RLN3 (Rn-Rln3-T1, cat. no. 1037211-T1, ACD), CCK (Rn-Cck-T2, cat. no. 532851-T2, ACD), vGAT1 (Rn-Slc32a1-T3, cat. no. 424541-T3, ACD), and round 2 (R2): CRHR1 (Rn-Crhr1-T4, cat. no. 318911-T4, ACD), vGlut2 (Rn-Slc17a6-T5, cat. no. 31701-T5, ACD) and TrkA (Rn-Ntrk1-T6, cat. no. 402611-T6, ACD).

Images of an ROI (1936 × 1460 pixels/220 × 166 µm, the same area for R1 and R2) located in NIc and NId were acquired for each slice, using an Axio Imager M2 fluorescence microscope (Zeiss) with an automatic z-stage, Axiocam 503 mono camera (Zeiss) and an EC Plan-Neofluar 40×/1.3 Oil M27 objective (scaling: 0.114 × 0.114 × 0.280 µm/pixel, respectively, in the x, y and z axis). The images were postprocessed using Zen (3.3 blue edition and 2.3 SP1 black edition, Zeiss), ImageJ and HiPlex Image Registration Software v1.0 (ACD), and cells were counted with an ImageJ Cell Counter plugin. If at least two unambiguous dots of specific fluorescence were detected within a cell boundary (identified by the presence of the DAPI-stained nucleus and/or a cell-shaped distribution of fluorescent mRNA signal), it was classified as expressing a specific mRNA molecule. A custom-made ImageJ macro was applied to estimate the area fraction of the ROI occupied by fluorescent dots representing CRHR1 and TrkA mRNA. The calculated area fraction was divided by the number of neurons expressing specific mRNA (TrkA or CRHR1) within a given ROI and expressed as mean area fraction per cell.

Statistical analysis

Cell counting, morphological and postsynaptic currents analyses were performed by experimenters blinded to the treatment conditions. The data were analyzed using Prism 8 (GraphPad Software, Boston, MA, USA) and R (R Foundation for Statistical Computing, Vienna, Austria) software. The normality of the data distribution and the homogeneity of variance were verified with the Shapiro–Wilk test and the Levene test. Data points identified as outliers using a ROUT test (Q = 1%) were excluded from the analysis. Data meeting the

criteria for the use of parametric tests were analyzed using unpaired t test (with Welch's correction, if the incorporated F test p value <0.05) or type III two-way ANOVA. Remaining data were analyzed using a Mann-Whitney test or type III two-way ANOVA with White's correction. After two-way ANOVA, Tukey's test (Prism) or Estimated Marginal Means (emmeans) function with Tukey adjustment method was used for post-hoc comparisons (R emmeans package), whenever necessary. Differences in the sag frequencies and proportions of c-Fos+ and c-Fos– neurons between groups were analyzed using Fisher's exact test. All data in graphs and tables are presented as mean ± SD or median ± IQR, depending on the tests used.

REFERENCES

1. Gugula A, Trenk A, Celary A, Cizio K, Tylko G, Blasiak A, Hess G (2022): Early-life stress modifies the reactivity of neurons in the ventral tegmental area and lateral hypothalamus to acute stress in female rats. *Neuroscience* 490: 49–65.

2. McReynolds JR, Christianson JP, Blacktop JM, Mantsch JR (2018): What does the Fos say? Using Fos-based approaches to understand the contribution of stress to substance use disorders. *Neurobiol Stress* 9: 271–285.

3. Schneider CA, Rasband WS, Eliceiri KW (2012): NIH Image to ImageJ: 25 years of image analysis. *Nat Methods* 9: 671–675.

4. Goto M, Swanson LW, Canteras NS (2001): Connections of the nucleus incertus. *J Comp Neurol* 438: 86–122.

5. Longair MH, Baker DA, Armstrong JD (2011): Simple neurite tracer: Open source software for reconstruction, visualization and analysis of neuronal processes. *Bioinformatics* 27: 2453–2454.

6. Sholl DA (1953): Dendritic organization in the neurons of the visual and motor cortices of the cat. *J Anat* 87: 387–406.

7. Scorcioni R, Polavaram S, Ascoli GA (2008): L-Measure: A web-accessible tool for the analysis, comparison and search of digital reconstructions of neuronal morphologies. *Nat Protoc* 3: 866–876.

8. Szlaga A, Sambak P, Trenk A, Gugula A, Singleton CE, Drwiega G, *et al.* (2022): Functional neuroanatomy of the rat nucleus incertus–medial septum tract: implications for the cell-specific control of the septohippocampal pathway. *Front Cell Neurosci* 16: 57.

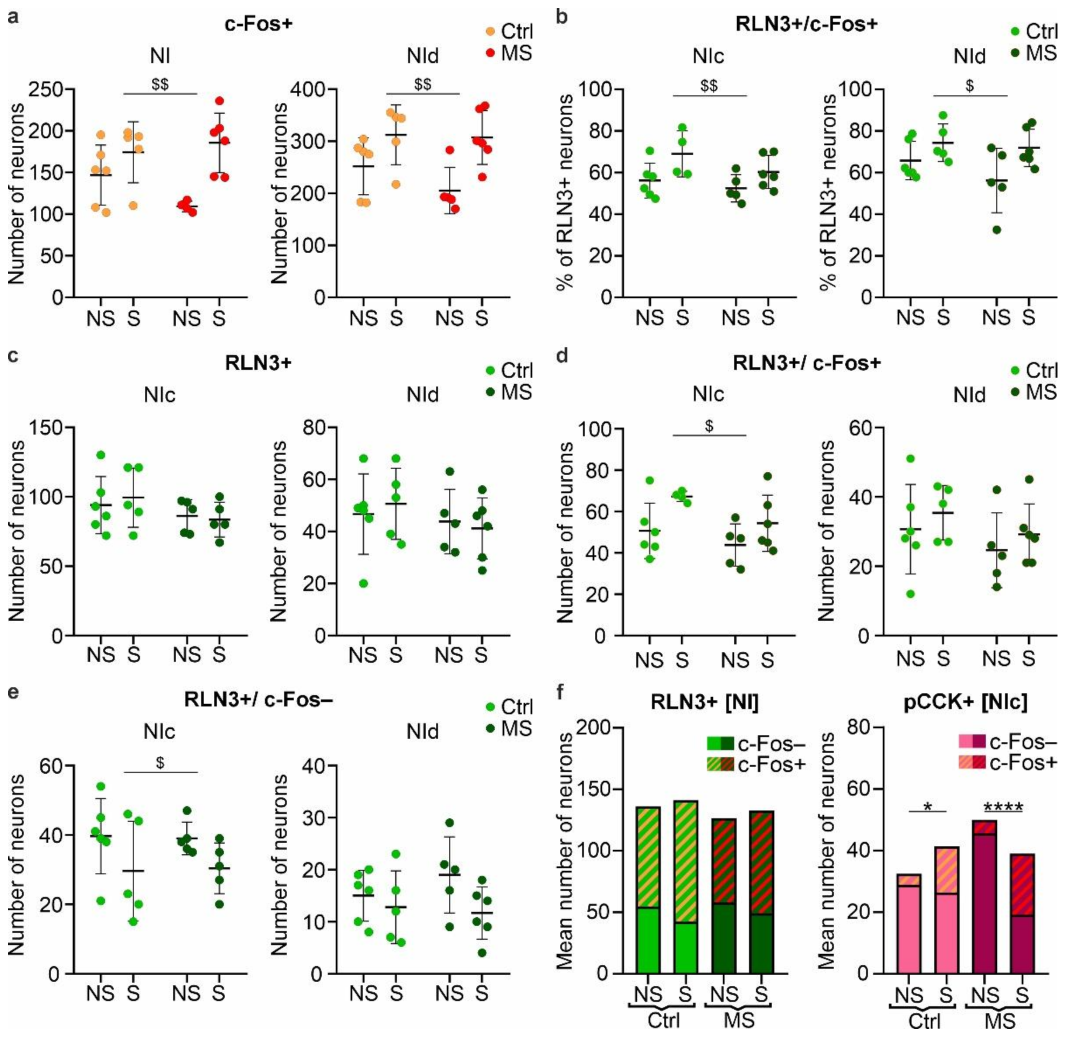

Supplementary Figure 1. Influence of restraint stress and MS on c-Fos expression in different NI areas. **(a)** The number of all NI and NId c-Fos+ cells was increased by restraint stress. **(b)** The proportion of c-Fos+ cells within the population of RLN3+ neurons in NIc and NId was increased by restraint stress. **(c)** Restraint stress and MS did not affect the number of RLN3+ NI and NId neurons. **(d)** Restraint stress increased the number of RLN3+/c-Fos+ cells in NIc but not NId. **(e)** The number of RLN3+/c-Fos– cells was decreased by restraint stress in NIc but not NId. Statistical significance in **(a-e)** was determined using two-way ANOVA: $ restraint stress effect. The number of each symbol indicates the level of statistical significance: * (p<0.05), ** (p<0.01). Please see **Supplementary Table 1** for details. **(f)** Proportions of RLN3-positive (RLN3+, left) and pCCK-positive (pCCK+, right) NI c-Fos+ and c-Fos– neurons in all tested groups of rats: control (Ctrl) and MS, subjected (S) and not subjected to restraint stress (NS). Note that both MS and restraint stress altered these proportions in pCCK+ but not RLN3+ cells. Statistical significance in **(f)** was determined using Fisher's exact test: * (p<0.05), **** (p<0.0001). Please see **Supplementary Table 2** for details. Abbreviations: NI, nucleus incertus; NIc, nucleus incertus pars compacta; NId, nucleus incertus pars dissipata; NS, not subjected to restraint stress; MS, maternal separation; pCCK, pro-cholecystokinin; RLN3, relaxin-3; S, subjected to restraint stress.

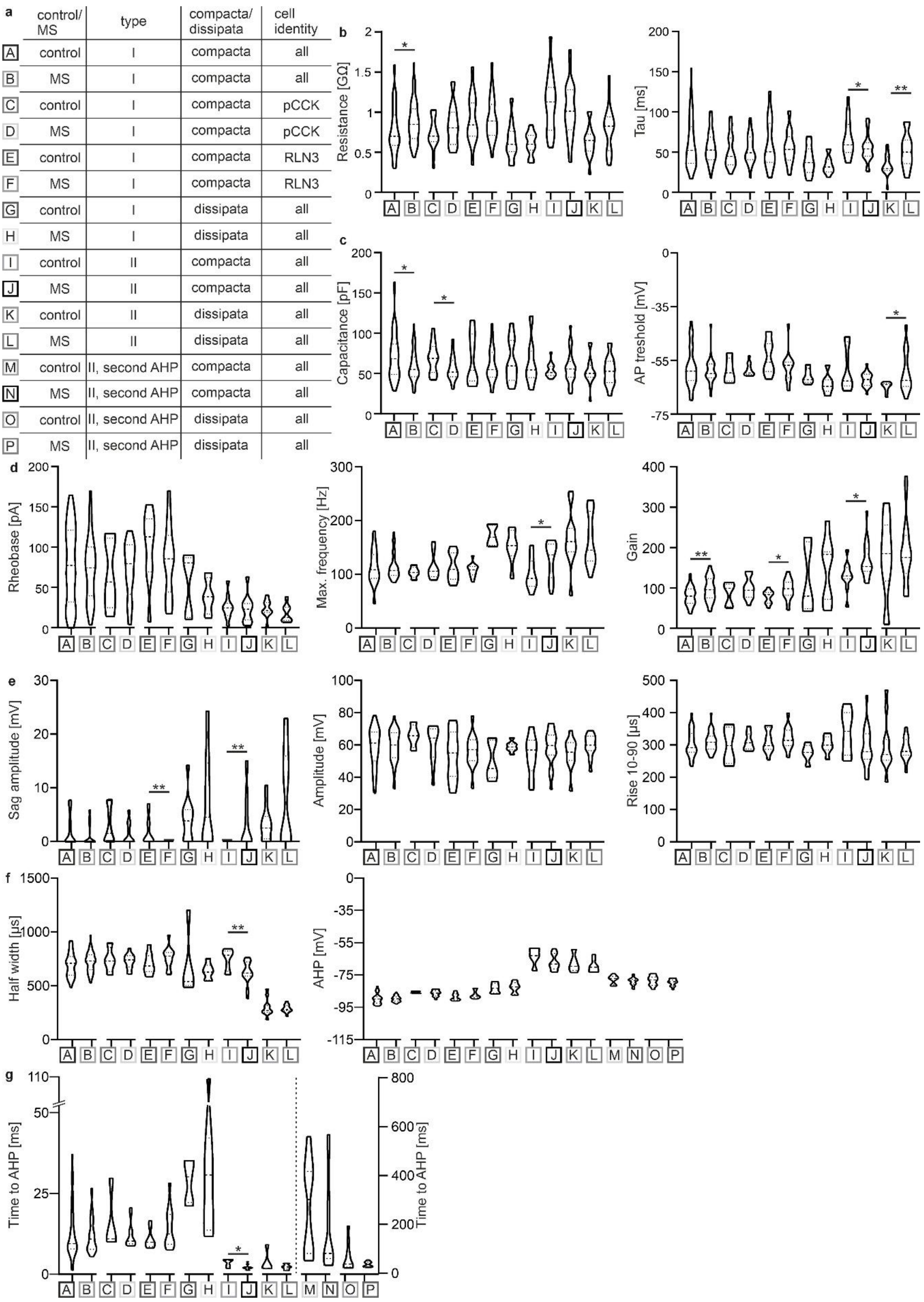

Supplementary Figure 2. Impact of MS on the properties of NI neurons. **(a)** The neuronal types compared. **(b-f)** Violin graphs comparing the membrane and spike properties of control and MS groups. **(g)** A violin graph comparing time to afterhyperpolarization of control and MS groups, left y axis refers to the A-L groups, right y axis refers to the M-P groups.

Asterisks represent statistical significance: * (p<0.05), ** (p<0.01), *** (p<0.001), **** (p<0.0001). Please see **Supplementary Table 7** for details.

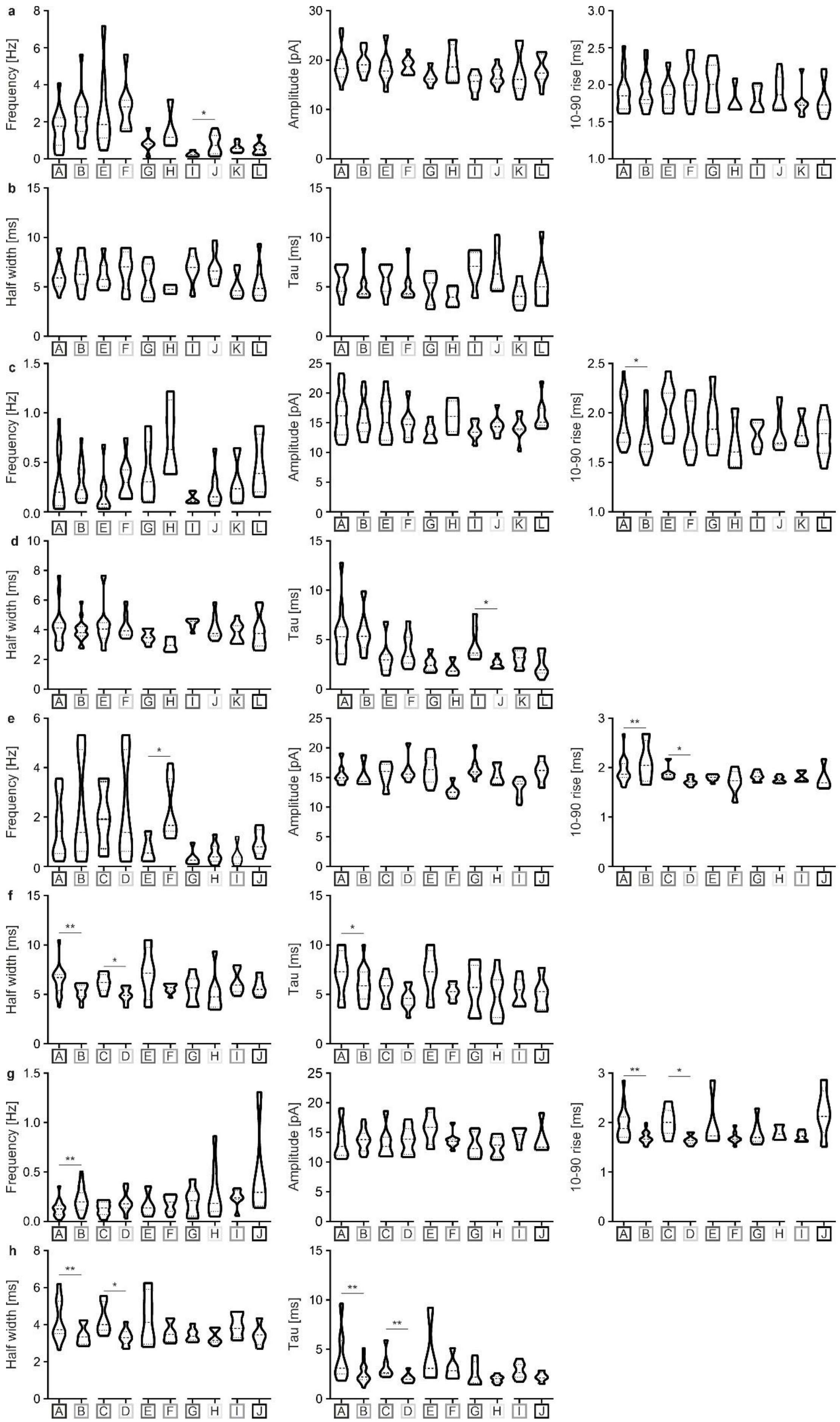
a
b
c
d
e
f
g
h
Frequency [Hz]
Amplitude [pA]
10-90 rise [ms]
Half width [ms]
Tau [ms]
A B E F G H I J K L
A B C D E F G H I J

Supplementary Figure 3. Impact of MS on the properties of the neural input to NI neurons. **(a, b)** Violin graphs comparing properties of outward spontaneous postsynaptic currents (sIPSCs) in control and MS groups. **(c, d)** Violin graphs comparing properties of inward spontaneous postsynaptic currents (sEPSCs) in control and MS groups. **(e, f)** Violin graphs comparing the properties of outward miniature postsynaptic currents (mIPSC) in control and MS groups. **(g, h)** Violin graphs comparing the properties of inward miniature postsynaptic currents (mEPSCs) in control and MS groups. Asterisks represents statistical significance: * ($p<0.05$), ** ($p<0.01$), *** ($p<0.001$), **** ($p<0.0001$). Please see **Supplementary Table 9** for details.

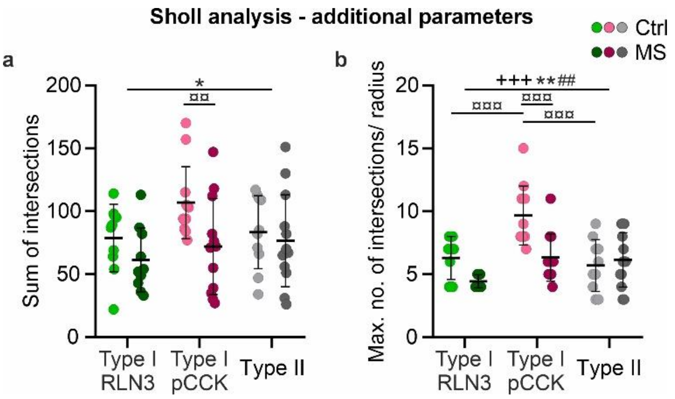

Supplementary Figure 4. Maternal separation selectively decreased the complexity of pCCK-positive NI neurons. **(a**, **b)** Additional parameters of the Sholl analysis of defined control and MS NI neurons: **(a)** sum of dendritic intersections with Sholl spheres; **(b)** maximal number of intersections per single sphere radius. Note the significantly higher complexity of type I pCCK-positive neurons, compared to type I RLN3-positive and type II NI neurons, and the MS-associated decrease. Statistical significance was determined using two-way ANOVA with a post-hoc Tukey test: * MS effect, + neuronal type effect, # interaction of MS and neuronal type, post-hoc: ¤. The number of each symbol indicates the level of statistical significance: * ($p<0.05$), ** ($p<0.01$), *** ($p<0.001$). Please see **Supplementary Table 10** for details.

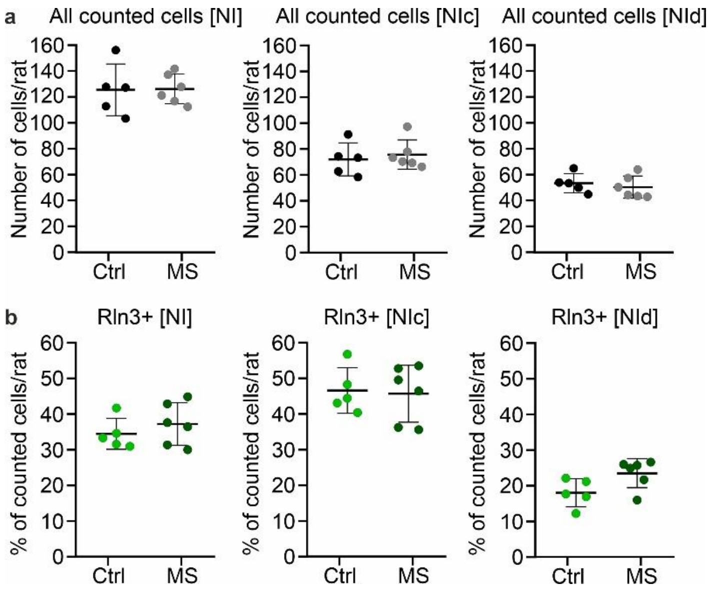

Supplementary Figure 5. Number of counted cells in control and MS rats in the RNAscope studies. **(a)** Total counted cells in the entire NI and its subregions, NIc and NId. **(b)** The percentage of counted cells expressing RLN3 mRNA in NI and its subregions. Statistical significance was assessed using a t-test, and revealed no significant differences.

Supplementary Table 1. c-Fos expression in NI.

Location	Mean ± SD (n)				Two-way ANOVA - effects (p and F values with df)			post hoc Tukey test (p, q and df values)			
	Ctrl		MS		Maternal separation	Restraint stress	Interaction	Ctrl NS vs. S NS	Ctrl NS vs. MS NS	MS NS vs. MS S	Ctrl S vs. MS S
	NS (6)	S (5)	NS (5)	S (6)							
The number of c-Fos+ cells											
NI	252 ± 55	312 ± 58	205 ± 44	307 ± 51	$F_{(1,18)}$ = 1.34, p = 0.26	$F_{(1,18)}$ = 13.11, **p = 0.002**	$F_{(1,18)}$ = 1.86, p = 0.37	-	-	-	-
NIc	105 ± 24	138 ± 23	78 ± 7	121 ± 29	$F_{(1,18)}$ = 4.81, **p = 0.04**	$F_{(1,18)}$ = 14.82, **p = 0.001**	$F_{(1,18)}$ = 0.25, p = 0.62	-	-	-	-
NId	147 ± 36	174 ± 37	109 ± 6 (4)	186 ± 36	$F_{(1,17)}$ = 0.81, p = 0.38	$F_{(1, 17)}$ = 12.73, **p = 0.002**	$F_{(1,17)}$ = 2.84, p = 0.11	-	-	-	-
The number of RLN3+ neurons											
NI	141 ± 29	150 ± 33	130 ± 18	137 ± 38	$F_{(1,17)}$ = 0.77, p = 0.39	$F_{(1,17)}$ = 0.38, p = 0.54	$F_{(1,17)}$ = 0.007, p = 0.94	-	-	-	-
NIc	94 ± 21	99 ± 21	86 ± 11	84 ± 13	$F_{(1,17)}$ = 2.41, p = 0.14	$F_{(1, 17)}$ = 0.03, p = 0.86	$F_{(1,17)}$ = 0.28, p = 0.61	-	-	-	-
NId	47 ± 15	51 ± 14	44 ± 12	41 ± 12	$F_{(1,18)}$ = 1.15, p = 0.30	$F_{(1,18)}$ = 0.01, p = 0.91	$F_{(1,18)}$ = 0.33, p = 0.57	-	-	-	-
The number of RLN3+/cFos+ neurons											
NI	81 ± 19	99 ± 15	69 ± 18 (4)	84 ± 18	$F_{(1,17)}$ = 6.21, **p = 0.02**	$F_{(1,17)}$ = 7.81, **p = 0.01**	$F_{(1,17)}$ = 0.15, p = 0.71	-	-	-	-
NIc	51 ± 13	63 ± 9 (4)	44 ± 10	54 ± 14	$F_{(1,17)}$ = 3.76, p = 0.07	$F_{(1, 17)}$ = 7.05, **p = 0.02**	$F_{(1,17)}$ = 0.35, p = 0.56	-	-	-	-
NId	31 ± 13	35 ± 8	25 ± 11	29 ± 9	$F_{(1,18)}$ = 1.92, p = 0.18	$F_{(1,18)}$ = 1.10, p = 0.31	$F_{(1,18)}$ = 0.0004, p = 0.99	-	-	-	-
The number of RLN3+/cFos- neurons											
NI	60 ± 4 (5)	42 ± 21	58 ± 11	41 ± 11 (5)	$F_{(1,16)}$ = 0.84, p = 0.78	$F_{(1,16)}$ = 8.66, **p = 0.01**	$F_{(1,16)}$ = 0.0003, p = 0.99	-	-	-	-
NIc	40 ± 11	30 ± 14	39 ± 5	38 ± 19 (5)	$F_{(1,17)}$ = 0.0002, p = 0.99	$F_{(1,17)}$ = 4.50, **p = 0.049**	$F_{(1,17)}$ = 0.03, p = 0.87	-	-	-	-
NId	15 ± 5	13 ± 7	19 ± 7	12 ± 5	$F_{(1,18)}$ = 0.31, p = 0.59	$F_{(1,18)}$ = 3.41, p = 0.08	$F_{(1,18)}$ = 0.99, p = 0.33	-	-	-	-
The percentage of c-Fos+ neurons in the RLN3+ population [%]											
NI	60 ± 6	71 ± 5	51 ± 5 (4)	64 ± 8	$F_{(1,17)}$ = 5.56, **p = 0.03**	$F_{(1,17)}$ = 12.62, **p = 0.002**	$F_{(1,17)}$ = 0.06, p = 0.80	-	-	-	-
NIc	56 ± 8	69 ± 11 (4)	52 ± 7	60 ± 8	$F_{(1,17)}$ = 2.79, p = 0.11	$F_{(1, 17)}$ = 7.75, **p = 0.01**	$F_{(1,17)}$ = 0.44, p = 0.52	-	-	-	-
NId	66 ± 9	74 ± 9	56 ± 15	72 ± 9	$F_{(1,18)}$ = 1.69, p = 0.21	$F_{(1,18)}$ = 6.87, **p = 0.02**	$F_{(1,18)}$ = 0.61, p = 0.47	-	-	-	-
The number of pCCK+ neurons											
NIc	4 ± 2	15 ± 9	4 ± 4	20 ± 9	$F_{(1,18)}$ = 1.78, p = 0.20	$F_{(1,18)}$ = 0.02, p = 0.88	$F_{(1,18)}$ = 5.89, p = 0.03	-	-	-	-
The number of pCCK+/cFos+ neurons											
NIc	4 ± 2	15 ± 9	4 ± 4	20 ± 9	$F_{(1,18)}$ = 0.90, p = 0.36	$F_{(1,18)}$ = 20.85, **p = 0.0002**	$F_{(1,18)}$ = 0.49, p = 0.49	-	-	-	-
The number of pCCK+/cFos- neurons											
NIc	29 ± 10	26 ± 11	46 ± 8	19 ± 9	$F_{(1,18)}$ = 1.39, p = 0.25	$F_{(1,18)}$ = 12.72, **p = 0.002**	$F_{(1,18)}$ = 8.79, **p = 0.008**	$q_{(18)}$ = 0.60, p = 0.97	$q_{(18)}$ = 0.60, **p = 0.04**	$q_{(18)}$ = 6.53, **p = 0.001**	$q_{(18)}$ = 1.79, p = 0.60
The percentage of c-Fos+ neurons in the pCCK+ population [%]											
NIc	13 ± 8	36 ± 25	8 ± 8	51 ± 20	$F_{(1,18)}$ = 0.46, p = 0.51	$F_{(1,18)}$ = 22.01, **p = 0.0002**	$F_{(1,18)}$ = 1.65, p = 0.21	-	-	-	-

The symbol (-) indicates no post-hoc analysis

Supplementary Table 2. Comparison of the proportions of c-Fos+ and c-Fos- neurons.

The number of c-Fos+ neurons/ total number of counted neurons								Fisher's exact test (p value)			
RLN3 (NI)				pCCK (NIc)				RLN3: Ctrl NS vs. S	RLN3: MS NS vs. S	pCCK: Ctrl NS vs. S	pCCK: MS NS vs. S
Ctrl NS	Ctrl S	MS NS	MS S	Ctrl NS	Ctrl S	MS NS	MS S				
81/136	99/141	69/127	84/133	4/33	15/41	4/50	20/39	0.08	0.17	**0.03**	**<0.0001**

Supplementary Table 3. Active, passive membrane properties and spike properties – comparison of pCCK+ and RLN3+ neurons.

Parameter	Mean/Median ± SD/iqr		test (p and t/U values with df)		
	C				
	pCCK (21)	RLN3 (15)	result	test	Welch's correction
resistance	737.4 ± 187.2	911.8 ± 310.1	$t_{(21.23)} = 1.94$, p = 0.07	t-test	yes
tau	50.81 ± 20.18	59.88 ± 30.66	$t_{(34)} = 1{,}072$, p = 0.29	t-test	no
capacitance	68.68 ± 32.34	53.88 ± 58.39	U = 140, p = 0.59	M-W	no
AP threshold	-43.68 ± 4.56	-38.66 ± 6.33	$t_{(14)} = 1.58$, p = 0.14	t-test	no
rheobase	6.57 ± 4.43	10.22 ± 4.27	$t_{(14)} = 1.57$, p = 0.14	t-test	no
max FQ	103.6 ± 10.46	113.1 ± 25.93	$t_{(14)} = 0.78$, p = 0.45	t-test	no
gain	85.47 ± 29.21	77.70 ± 15.88	$t_{(14)} = 0.70$, p = 0.50	t-test	no
sag amplitude	1.52 ± 6.23	0 ± 3.35 (13)	U = 105, p = 0.23	M-W	no
amplitude	65.50 ± 7.36	53.37 ± 14.96	$t_{(13)} = 1.53$, p = 0.15	t-test	no
rise 10-90	298.9 ± 58.78	310.8 ± 35.59	$t_{(13)} = 0.49$, p = 0.64	t-test	no
half width	738.9 ± 121.8	701.3 ± 95.16 (14)	$t_{(13)} = 0.62$, p = 0.55	t-test	no
AHP	-70.80 ± 0.67	-73.60 ± 2.174	$t_{(13)} = 2.48$, **p = 0.0278**	t-test	no
time to AHP	11.02 ± 15.22	9.97 ± 4.47 (13)	U = 8, p = 0.15	M-W	no

Supplementary Table 4. Spontaneous and miniature postsynaptic currents properties – comparison of pCCK+ and RLN3+ neurons.

Parameter	Mean/Median ± SD/iqr		test (p and t/U values with df)		
	C (n)				
	pCCK (7)	RLN3 (4)	result	test	Welch's correction
mIPSC fq	2.00 ± 1.22	0.68 ± 0.55	$t_{(9)} = 2.01$, $p = 0.08$	t-test	no
mIPSC amplitude	15.56 ± 6.56	14.34 ± 4.88	$U = 7$, $p = 0.23$	M-W	no
mIPSP 10-90 rise	1.86 ± 0.42	2.05 ± 1.03	$U = 9$, $p = 0.41$	M-W	no
mIPSP half width	6.14 ± 0.89	7.13 ± 2.80	$t_{(3.353)} = 0.68$, $p = 0.54$	t-test	yes
mIPSP tau	5.59 ± 1.44	7.07 ± 2.62	$t_{(9)} = 1.23$, $p = 0.25$	t-test	no
mEPSP fq	0.13 ± 0.07	0.17 ± 0.13	$t_{(9)} = 0.73$, $p = 0.48$	t-test	no
mEPSP amplitude	13.22 ± 2.74	15.76 ± 2.83	$t_{(9)} = 1.46$, $p = 0.18$	t-test	no
mEPSP 10-90 rise	2.00 ± 0.81	1.73 ± 1.22	$U = 10$, $p = 0.53$	M-W	no
mEPSP half width	4.27 ± 0.83	4.33 ± 1.55	$t_{(9)} = 0.08$, $p = 0.94$	t-test	no
mEPSP tau	3.22 ± 1.28	4.38 ± 3.30	$U = 14$, $p > 0.99$	t-test	no

Supplementary Table 5. Active, passive membrane properties and spike properties – two-way ANOVA analysis.

Parameter	Mean ± SD				Two-way ANOVA - effects (p and F values with df)				post hoc Tukey test (p, t and df values)			
	C		MS									
	Type I (n)	Type II (n)	Type I (n)	Type II (n)	Maternal separation	Type	Interaction	White adjustment	I C vs. I MS	I C vs. II C	II C vs. II MS	I MS vs. II MS
resistance	751.9 ± 287.2 (89)	868.0 ± 374.1 (52)	794.4 ± 272.3 (105)	887.9 ± 336.9 (59)	$F_{(1,301)}$ = 0.72, p = 0.40	$F_{(1,301)}$ = 8.10, **p = 0.0047**	$F_{(1,301)}$ = 0.09, p = 0.76	no	-	-	-	-
tau	52.17 ± 25.30 (89)	45.50 ± 21.16 (52)	45.61 ± 19.53 (105)	48.22 ± 24.03 (59)	$F_{(1,296)}$ = 0.50, p = 0.48	$F_{(1,296)}$ = 0.56, p = 0.46	$F_{(1,301)}$ = 2.92, p = 0.09	no	-	-	-	-
capacitance	70.95 ± 27.89 (89)	52.49 ± 14.62 (52)	59.67 ± 20.80 (105)	54.58 ± 18.69 (59)	$F_{(1,296)}$ = 3.00, p = 0.08	$F_{(1,296)}$ = 19.66, **p < 0.0001**	$F_{(1,296)}$ = 6.35, **p = 0.0123**	no	$t_{(296)}$ = 3.55, **p = 0.0027**	$t_{(296)}$ = 4.72, **p < 0.0001**	$t_{(296)}$ = 0.49, p = 0.99	$t_{(296)}$ = 1.42, p = 0.64
AP threshold	-42.89 ± 6.88 (45)	-45.44 ± 7.337 (30)	-43.99 ± 6.58 (65)	-45.27 ± 6.19 (46)	$F_{(1,176)}$ = 2.82, p = 0.10	$F_{(1,176)}$ = 4.88, **p = 0.0285**	$F_{(1,176)}$ = 0.40, p = 0.53	no	-	-	-	-
rheobase	75.80 ± 47.47 (42)	24.47 ± 12.39 (23)	69.03 ± 39.70 (53)	20.51 ± 14.12 (41)	$F_{(1,301)}$ = 0.87, p = 0.35	$F_{(1,301)}$ = 75.3, **p < 0.0001**	$F_{(1,301)}$ = 0.06, p = 0.81	no	-	-	-	-
max FQ	120.9 ± 34.90 (45)	128.6 ± 51.33 (30)	123.3 ± 33.02 (65)	142.9 ± 43.60 (46)	$F_{(1,301)}$ = 13.10, p = 0.32	$F_{(1,301)}$ = 0.01, p = 0.16	$F_{(1,301)}$ = 0.42, p = 0.27	yes	-	-	-	-
gain	90.3 ± 43.48 (43)	153.2 ± 75.91 (53)	103.4 ± 42.15 (23)	176.9 ± 61.72 (40)	$F_{(1,155)}$ = 3.60, p = 0.11	$F_{(1,155)}$ = 79.40, **p < 0.0001**	$F_{(1,155)}$ = 0.44, p = 0.69	yes	-	-	-	-
sag amplitude	6.00 ± 3.71 (44)	4.30 ± 2.92 (17)	6.18 ± 3.64 (34)	11.87 ± 7.56 (27)	$F_{(1,115)}$ = 17.24, **p < 0.0001**	$F_{(1,115)}$ = 4.56, **p = 0.0349**	$F_{(1,115)}$ = 15.64, **p = 0.0001**	no	$t_{(115)}$ = 0.23, p = 0.99	$t_{(115)}$ = 1.73, p = 0.61	$t_{(115)}$ = 7.12, **p < 0.0001**	$t_{(115)}$ = 6.46, **p < 0.0001**
amplitude	57.54 ± 12.48 (40)	54.27 ± 10.80 (52)	59.41 ± 9.26 (20)	58.63 ± 9.33 (37)	$F_{(1,144)}$ = 2.92, p = 0.09	$F_{(1,144)}$ = 1.23, p = 0.27	$F_{(1,144)}$ = 0.47, p = 0.50	no	-	-	-	-
rise 10-90	300.1 ± 39.80 (40)	315.2 ± 76.12 (52)	308.5 ± 28.80 (21)	289.0 ± 51.99 (37)	$F_{(1,143)}$ = 1.21, p = 0.41	$F_{(1,143)}$ = 0.07, p = 0.84	$F_{(1,143)}$ = 4.53, p = 0.11	yes	-	-	-	-
half width	679.3 ± 116.2 (40)	486.5 ± 242.3 (52)	710.4 ± 95.41 (21)	508.9 ± 209.2 (37)	$F_{(1,141)}$ = 0.89, p = 0.45	$F_{(1,141)}$ = 48.70, **p < 0.0001**	$F_{(1,141)}$ = 0.02, p = 0.90	yes	-	-	-	-
AHP	-71.28 ± 3.730 (40)	-63.16 ± 2.85 (52)	-71.14 ± 2.60 (21)	-64.28 ± 2.23 (36)	$F_{(1,146)}$ = 0.95, p = 0.33	$F_{(1,146)}$ = 224.30, **p < 0.0001**	$F_{(1,146)}$ = 1.57, p = 0.21	no	-	-	-	-
time to AHP	17.41 ± 10.69 (40)	152.9 ± 151.4 (52)	13.96 ± 5.61 (21)	72.65 ± 54.15 (37)	$F_{(1,128)}$ = 0.06, p = 0.08	$F_{(1,128)}$ = 60.00, **p < 0.0001**	$F_{(1,128)}$ = 11.61, **p = 0.0008**	yes	$t_{(128)}$ = 0.25, p = 0.99	$t_{(128)}$ = -7.75, **p < 0.0001**	$t_{(128)}$ = 4.35, **p = 0.0002**	$t_{(128)}$ = -3.94, **p = 0.0008**

The symbol (-) indicates no post-hoc analysis

Supplementary Table 6. Sag occurrence in pCCK+ and RLN3+ neurons.

Number of sag+ neurons/total nuomber of neurons						Fisher's exact test (p value)				
C		C		MS						
pCCK	RLN3	I	II	I	II	pCCK vs. RLN3	I C vs. I MS	I C vs. II C	I MS vs. II MS	II MS vs. II C
11/21	6/15	46/89	22/46	38/92	36/59	0.52	0.18	0.72	0.24	**0.0203**

Supplementary Table 7. Active, passive membrane properties and spike properties – t-test.

Parameter	Mean/Median ± SD/iqr, group (n)																t-test (p and t/U values with df)							
	A (72)	B (72)	C (21)	D (22)	E (15)	F (27)	G (12)	H (16)	I (19)	J (30)	K (17)	L (20)	M	N	O	P	A vs. B	C vs. D	E vs. F	G vs. H	I vs. J	K vs. L	M vs. N	O vs. P
resistance	700.3 ± 378.6	874.9 ± 368.1	737.4 ± 187.2	835.5 ± 247.1	911.8 ± 310.1	915.7 ± 268.4	642.4 ± 232.1	611.8 ± 144.4 (15)	1109 ± 356.2	1016 ± 324.9	635 ± 193.9	792.4 ± 278.4	-	-	-	-	U = 1989, **p = 0.0158**, M-W	$t_{(41)}$ = 1.46, p = 0.15, t-test	$t_{(40)}$ = 0.04, p = 0.97, t-test	$t_{(25)}$ = 0.42, p = 0.68, t-test	$t_{(47)}$ = 0.94, p = 0.35, t-test	$t_{(35)}$ = 1.96, p = 0.06, t-test	-	-
tau	52.52 ± 45.23	52.96 ± 28.21 (71)	50.81 ± 20.18	53.29 ± 18.15	59.88 ± 30.66	56.17 ± 20.20 (26)	39.90 ± 17.12	34.06 ± 10.73 (13)	52.17 ± 25.30	54.95 ± 16.70 (29)	33.96 ± 12.83	51.41 ± 20.61	-	-	-	-	U = 2544, p = 0.9631, M-W	$t_{(41)}$ = 0.42, p = 0.67, t-test	$t_{(39)}$ = 0.47, p = 0.64, t-test	$t_{(23)}$ = 1.03, p = 0.31, t-test	$t_{(46)}$ = 2.03, **p = 0.0484**, t-test	$t_{(35)}$ = 3.02, **p = 0.0046**, t-test	-	-
capacitance	65.94 ± 37.5	60.49 ± 24.37 (71)	68.81 ± 18.33	55.88 ± 15.03	53.88 ± 58.39	54.82 ± 30.61 (26)	64.62 ± 26.22	62.43 ± 26.74	51.16 ± 11.61 (16)	55.59 ± 19.64	53.00 ± 16.66	52.68 ± 17.57	-	-	-	-	U =1987, **p = 0.0214,** M-W	$t_{(41)}$ = 2.54, **p = 0.0152**, t-test	U = 185, p = 0.80, M-W	$t_{(26)}$ = 0.22, p = 0.83, t-test	U = 233, p = 0.88, M-W	$t_{(35)}$ = 0.06, p = 0.95, t-test	-	-
AP threshold	-43.95 ± 8.6 (38)	44.82 ± 5.19 (42)	-44.60 ± 8.57 (5)	-44.66 ± 3.78 (12)	-40.67 ± 10.31 (11)	-41.84 ± 5.85 (5)	-45.89 ± 2.90 (5)	-48.68 ± 3.66 (7)	-47.68 ± 16.40 (11)	-47.14 ± 4.17 (22)	-49.78 ± 1.81 (11)	-44.51 ± 7.40 (17)	-	-	-	-	U = 768, p = 0.7756, M-W	U = 28, p = 0.88, M-W	U = 60, p = 0.26, M-W	$t_{(10)}$ = 1.41, p = 0.19, t-test	U = 112, p = 0.75, M-W	$t_{(18.85)}$ = 2.81, **p = 0.0113**, Welch	-	-
rheobase	78.50 ± 48.25 (37)	73.66 ± 39.97 (46)	65.67 ± 44.27 (5)	71.58 ± 35.18 (13)	102.20 ± 42.71 (11)	82.60 ± 45.16 (15)	80.41 ± 74.31 (5)	38.45 ± 45.01 (7)	24.57 ± 15.55 (11)	22.96 ± 20.34 (24)	21.21 ± 12.13 (12)	12.36 ± 16.25 (17)	-	-	-	-	$t_{(81)}$ = 0.5, p = 0.6181, t-test	$t_{(16)}$ = 0.2978, p = 0.7697, t-test	$t_{(24)}$ = 1.118, p = 0.2748, t-test	U = 13, p = 0.5303, M-W	U = 117.5, p = 0.6177, M-W	U = 70, p = 0.1620, M-W	-	-
max FQ	108.9 ± 44.12 (38)	107.4 ± 25.39 (45)	103.3 ± 18.65 (5)	105.6 ± 33.09 (13)	113.1 ± 25.93 (11)	107.5 ± 12.15 (14)	172.0 ± 18.84 (5)	151.2 ± 31.95 (7)	92.27 ± 47.19 (11)	136.1 ± 62.32 (24)	165.8 ± 50.66(12)	163.6 ± 47.81 (17)	-	-	-	-	U = 847, p = 0.9456, M-W	U = 129, p = 0.63, M-W	$t_{(13.44)}$ = 0.66, p = 0.52, Welch	$t_{(10)}$ = 1.29, p = 0.23, t-test	U = 74, **p = 0.0397**, M-W	$t_{(27)}$ = 0.12, p = 0.90, t-test	-	-
gain	80.30 ± 23.50 (36)	97.09 ± 29.27 (46)	85.47 ± 29.21 (5)	98.96 ± 26.14 (13)	77.70 ± 15.88 (11)	96.87 ± 25.23 (15)	121.10 ± 86.57 (5)	144.70 ± 81.69 (7)	129.70 ± 28.20 (11)	153.20 ± 56.50 (23)	174.60 ± 96.85 (12)	189.60 ± 81.26 (17)	-	-	-	-	$t_{(80)}$ = 2.81, **p = 0.0063**, t-test	$t_{(16)}$ = 0.95, p = 0.36, t-test	$t_{(24)}$ = 2.21, **p = 0.0367**, t-test	$t_{(10)}$ = 0.48, p = 0.64, t-test	U = 60.50, **p = 0.0138**, M-W	$t_{(27)}$ = 0.45, p = 0.65, t-test	-	-
sag amplitude	0 ± 3.604 (54)	0 ± 0.009 (63)	1.52 ± 6.23 (21)	0 ± 3.32 (21)	0 ± 3.36 (13)	0 ± 0 (21)	3.84 ± 5.92 (12)	4.52 ± 14.62 (16)	0 ± 0 (15)	0 ± 6.45 (26)	2.51 ± 4.15 (17)	7.20 ± 15.91 (20)	-	-	-	-	U = 1436, p = 0.0845, M-W	U = 179, p = 0.26, M-W	U = 94.50, **p = 0.0154**, M-W	U = 80, p = 0.46, M-W	U = 112.5, **p = 0.0054**, M-W	U = 133, p = 0.26, M-W	-	-
	A (35)	B (45)	C (4)	D (13)	E (11)	F (15)	G (5)	H (7)	I (10)	J (22)	K (11)	L (15)	M (5)	N (11)	O (8)	P (15)								
amplitude	58.59 ± 12.34	59.54 ± 10.0 (44)	65.50 ± 7.36	60.60 ± 11.43	53.37 ± 14.96	56.29 ± 10.14	50.18 ± 12.09	58.95 ± 3.26	53.44 ± 12.35 (9)	58.14 ± 10.68	54.94 ± 9.93	59.39 ± 7.00 (14)	-	-	-	-	$t_{(77)}$ = 0.376, p = 0.7077, t-test	$t_{(15)}$ = 0.80, p = 0.44, t-test	$t_{(24)}$ = 0.59, p = 0.56, t-test	$t_{(4.42)}$ = 1.58, p = 0.18, Welch	$t_{(29)}$ = 1.07, p = 0.306, t-test	$t_{(23)}$ = 1.31, p = 0.20, t-test	-	-
rise 10-90	303.6 ± 40.27	309.6 ± 29.2	298.9 ± 58.78	309.9 ± 26.01	310.8 ± 35.59	321.0 ± 34.31	275.4 ± 28.10	301.1 ± 26.92	338.6 ± 66.77 (8)	290.7 ± 60.67	298.2 ± 80.96	286.2 ± 36.30 (14)	-	-	-	-	$t_{(59,8)}$ = 0.74, p = 0.462, Welch	$t_{(3.369)}$ = 0.36, p = 0.74, Welch	$t_{(24)}$ = 0.73, p = 0.47, t-test	$t_{(10)}$ = 1.60, p = 0.14, t-test	$t_{(28)}$ = 1.86, p = 0.07, t-test	$t_{(13.16)}$ = 0.45, p = 0.65, Welch	-	-
half width	695.1 ± 111.0 (34)	722.4 ± 93.59 (44)	738.9 ± 121.80	727.1 ± 67.69	701.3 ± 95.16 (10)	758.9 ± 97.61 (14)	539.8 ± 416.80	626.7 ± 200.50	784.9 ± 198.50 (9)	618.4 ± 64.70	612.4 ± 279.40	601.9 ± 69.00	-	-	-	-	$t_{(35)}$ = 0.15, p = 0.88, t-test	$t_{(15)}$ = 0.25, p = 0.80, t-test	$t_{(22)}$ = 1.44, p = 0.16, t-test	U = 11, p = 0.34, M-W	U = 32, **p = 0.0072**, M-W	U = 76, p = 0.76, M-W	-	-
AHP	-71.80 ± 4.78	-71.60 ± 3.14	-70.80 ± 0.67	-71.29 ± 1.95	-74.62 ± 3.75	-72.08 ± 4.10	-68.23 ± 3.23	-67.73 ± 3.48	-48.27 ± 5.70	-62.84 ± 2.66	-52.19 ± 4.91	-64.07 ± 2.37	-52.81 ± 5.54	-63.46 ± 3.12	-54.50 ± 3.94	-64.58 ± 2.06	U = 722, p = 0.5289, M-W	$t_{(15)}$ = 0.48, p = 0.64, t-test	U = 55, p = 0.16, M-W	$t_{(10)}$ = 0.25, p = 0.81, t-test	$t_{(16)}$ = 1.46, p = 0.16, t-test	$t_{(30)}$ = 1.31, p = 0.20, t-test	$t_{(11)}$ = 0.65, p = 0.53, t-test	$t_{(24)}$ = 1.11, p = 0.28, t-test
	A (35)	B (45)	C (4)	D (13)	E (11)	F (15)	G (5)	H (7)	I (10)	J (22)	K (11)	L (15)	M (10)	N (22)	O (11)	P (15)								
time to AHP	11.42 ± 9.901 (33)	12.59 ± 7.581 (41)	11.02 ± 15.22	10.35 ± 3.97 (12)	10.90 ± 2.94 (9)	14.34 ± 6.10 (14)	30.17 ± 12.76	30.79 ± 28.49	4.36 ± 2.26	2.07 ± 0.45	1.94 ± 4.39	2.37 ± 1.50	306.30 ± 335.97	85.61 ± 227.82	41.52 ± 73.58 (10)	39.69 ± 19.16 (12)	U = 657, p = 0.8373, M-W	U = 16, p = 0.38, M-W	$t_{(19.89)}$ = 1.81, p = 0.09, Welch	U = 16, p = 0.88, M-W	U = 12, **p = 0.0460**, M-W	U = 20, p > 0.99, M-W	U = 74, p = 0.15, M-W	U = 58, p = 0.92, M-W

The symbol (-) indicates no analysis

Supplementary Table 8. Spontaneous and miniature postsynaptic currents properties – two-way ANOVA analysis.

Parameter	Mean ± SD				Two-way ANOVA - effects (p and F values with df)				post hoc Tukey test (p, t and df values)			
	C		MS		Maternal separation	Type	Interaction	White adjustment	I C vs. I MS	I C vs. II C	II C vs. II MS	I MS vs. II MS
	Type I (n)	Type II (n)	Type I (n)	Type II (n)								
sIPSC fq	1.35 ± 0.96 (21)	0.45 ± 0.28 (14)	2.10 ± 1.19 (20)	0.78 ± 0.59 (18)	$F_{(1,69)}$ = 6.54, p = 0.11	$F_{(1,69)}$ = 27.92, p < **0.0001**	$F_{(1,69)}$ = 1.00, p = 0.52	yes	-	-	-	-
sIPSP amplitude	18.09 ± 3.08 (22)	16.50 ± 3.54 (14)	19.12 ± 2.58 (21)	17.32 ± 2.49 (18)	$F_{(1,71)}$ = 1.84, p = 0.18	$F_{(1,71)}$ = 6.18, **p = 0.0153**	$F_{(1,71)}$ = 0.03, p = 0.87	no	-	-	-	-
sIPSP 10-90 rise	1.92 ± 0.27 (22)	1.78 ± 0.18 (14)	1.89 ± 0.24 (21)	1.84 ± 0.23 (18)	$F_{(1,71)}$ = 0.07, p = 0.79	$F_{(1,71)}$ = 2.82, p = 0.10	$F_{(1,71)}$ = 0.90, p = 0.34	no	-	-	-	-
sIPSP half width	5.96 ± 1.47 (22)	5.81 ± 1.64 (14)	6.08 ± 1.54 (21)	6.13 ± 1.79 (18)	$F_{(1,71)}$ = 0.34, p = 0.56	$F_{(1,71)}$ = 0.02, p = 0.90	$F_{(1,71)}$ = 0.07, p = 0.79	no	-	-	-	-
sIPSP tau	5.21 ± 1.43 (22)	5.30 ± 1.96 (14)	5.23 ± 1.61 (21)	5.92 ± 2.26 (18)	$F_{(1,71)}$ = 0.56, p = 0.46	$F_{(1,71)}$ = 0.85, p = 0.36	$F_{(1,71)}$ = 0.49, p = 0.49	no	-	-	-	-
sEPSP fq	0.32 ± 0.29 (21)	0.19 ± 0.15 (13)	0.33 ± 0.28 (20)	0.31 ± 0.24 (17)	$F_{(1,60)}$ = 0.20, p = 0.66	$F_{(1,60)}$ = 1.93, p = 0.17	$F_{(1,60)}$ = 0.21, p = 0.65	no	-	-	-	-
sEPSP amplitude	15.50 ± 3.54 (22)	14.01 ± 1.84 (14)	15.70 ± 2.92 (21)	15.36 ± 2.24 (18)	$F_{(1,71)}$ = 1.39, p = 0.24	$F_{(1,71)}$ = 1.94, p = 0.17	$F_{(1,71)}$ = 0.77, p = 0.38	no	-	-	-	-
sEPSP 10-90 rise	1.90 ± 0.26 (22)	1.80 ± 0.14 (14)	1.74 ± 0.23 (21)	1.79 ± 0.20 (18)	$F_{(1,71)}$ = 2.96, p = 0.09	$F_{(1,71)}$ = 0.36, p = 0.55	$F_{(1,71)}$ = 2.23, p = 0.14	no	-	-	-	-
sEPSP half width	3.85 ± 0.89 (21)	4.07 ± 0.64 (14)	3.76 ± 0.78 (21)	4.01 ± 0.98 (18)	$F_{(1,70)}$ = 0.15, p = 0.70	$F_{(1,70)}$ = 1.42, p = 0.24	$F_{(1,70)}$ = 0.01, p = 0.93	no	-	-	-	-
sEPSP tau	2.60 ± 0.86 (19)	3.45 ± 1.04 (14)	3.00 ± 1.36 (20)	2.50 ± 0.87 (16)	$F_{(1,65)}$ = 1.11, p = 0.30	$F_{(1,65)}$ = 0.43, p = 0.51	$F_{(1,65)}$ = 6,57, **p = 0.0127**	no	$t_{(65)}$ = 1.65, p = 0.65	$t_{(65)}$ = 3.10, p = 0.14	$t_{(65)}$ = 3.35, p = 0.09	$t_{(65)}$ = 1.99, p = 0.50
mIPSC fq	1.30 ± 1.19 (25)	0.65 ± 0.31 (7)	1.29 ± 1.15 (30)	0.97 ± 0.53 (5)	$F_{(1,60)}$ = 0.20, p = 0.66	$F_{(1,60)}$ = 1.93, p = 0.17	F(1,60) = 0.21, p = 0.65	no	-	-	-	-
mIPSC amplitude	14.97 ± 2.17 (25)	15.39 ± 1.54 (7)	15.36 ± 2.34 (30)	16.02 ± 1.97 (5)	$F_{(1,63)}$ = 0.52, p = 0.47	$F_{(1,63)}$ = 0.59, p = 0.45	$F_{(1,63)}$ = 0.03, p = 0.86	no	-	-	-	-
mIPSP 10-90 rise	1.87 ± 0.26 (25)	1.76 ± 0.07 (7)	1.78 ± 0.12 (30)	1.81 ± 0.11 (5)	$F_{(1,63)}$ = 0.22, p = 0.64	$F_{(1,63)}$ = 0.54, p = 0.46	$F_{(1,63)}$ = 1.79, p = 0.19	no	-	-	-	-
mIPSP half width	6.15 ± 1.51 (25)	6.17 ± 1.18 (7)	5.10 ± 0.84 (30)	5.78 ± 0.97 (5)	$F_{(1,62)}$ = 3.54, p = 0.06	$F_{(1,62)}$ = 0.85, p = 0.36	$F_{(1,62)}$ = 0.73, p = 0.40	no	-	-	-	-
mIPSP tau	5.83 ± 1.83 (25)	5.25 ± 1.41 (6)	4.99 ± 1.47 (30)	5.05 ± 1.74 (5)	$F_{(1,63)}$ = 0.97, p = 0.33	$F_{(1,63)}$ = 0.24, p = 0.63	$F_{(1,63)}$ = 0.37, p = 0.55	no	-	-	-	-
sEPSP fq	0.16 ± 0.10 (24)	0.24 ± 0.09 (6)	0.22 ± 0.13 (30)	0.47 ± 0.48 (5)	$F_{(1,61)}$ = 7.82, **p = 0.0069**	$F_{(1,61)}$ = 9.93, **p = 0.0025**	$F_{(1,61)}$ = 2.06, p = 0.16	no	-	-	-	-
sEPSP amplitude	13.36 ± 2.64 (25)	14.43 ± 1.37 (7)	13.37 ± 1.78 (30)	13.89 ± 2.61 (5)	$F_{(1,63)}$ = 0.15, p = 0.70	$F_{(1,63)}$ = 1.28, p = 0.26	$F_{(1,63)}$ = 0.15, p = 0.70	no	-	-	-	-
sEPSP 10-90 rise	1.87 ± 0.28 (23)	1.72 ± 0.10 (7)	1.72 ± 0.12 (30)	1.65 ± 0.11 (5)	$F_{(1,61)}$ = 2.95, p = 0.09	$F_{(1,61)}$ = 3.08, p = 0.08	$F_{(1,61)}$ = 0.33, p = 0.57	no	-	-	-	-
sEPSP half width	4.13 ± 1.00 (25)	3.95 ± 0.61 (7)	3.43 ± 0.53 (30)	3.43 ± 0.61 (5)	$F_{(1,63)}$ = 6.24, **p = 0.0151**	$F_{(1,63)}$ = 0.14, p = 0.71	$F_{(1,63)}$ = 0.13, p = 0.72	no	-	-	-	-
sEPSP tau	3.18 ± 1.39 (22)	2.82 ± 0.84 (5)	2.43 ± 0.94 (30)	2.20 ± 0.50 (5)	$F_{(1,58)}$ = 3.74, p = 0.06	$F_{(1,58)}$ = 0.69, p = 0.41	$F_{(1,58)}$ = 0.04, p = 0.84	no	-	-	-	-

The symbol (-) indicates no post-hoc analysis

Supplementary Table 10. Sholl analysis - additional parameters.

Parameter	I RLN3 - mean ± SD (n)		I pCCK- mean ± SD (n)		II - mean ± SD (n)		Two-way ANOVA (type III) - effects (p and F values with df)			Post hoc Tukey test (p and t values with df)								
										Ctrl			MS			Crtl vs. MS		
	Ctrl (10)	MS (10)	Ctrl (12)	MS (12)	Ctrl (10)	MS (13)	Maternal separation	Type	Interaction	I RLN3 vs. I pCCK	I RLN3 vs. II	I pCCK vs. II	I RLN3 vs. I pCCK	I RLN3 vs. II	I pCCK vs. II	I RLN3	I pCCK	II
Sum of intersections	77.8 ± 26.9	60.3 ± 25.5	105.8 ± 28.5	71.0 ± 38.1	82.4 ± 29.0	75.6 ± 36.5	$F_{(1,61)} = 6.44$, **p = 0.01**	$F_{(2,61)} = 2.05$, p = 0.14	$F_{(2,61)} = 1.17$, p = 0.32	$t_{(61)} = 2.07$, p = 0.10	$t_{(61)} = -0.33$, p = 0.94	$t_{(61)} = 1.73$, p = 0.20	$t_{(61)} = 0.79$, p = 0.71	$t_{(61)} = -1.15$, p = 0.49	$t_{(61)} = -0.37$, p = 0.93	$t_{(61)} = 0.81$, p = 0.42	$t_{(61)} = 2.70$, **p = 0.009**	$t_{(61)} = 0.51$, p = 0.61
Max. no. of intersections/ radius	6.3 ± 1.7	4.9 ± 1.5	9.7 ± 2.4	6.3 ± 1.9	5.7 ± 2.1	6.2 ± 2.2	$F_{(1,61)} = 8.56$, **p = 0.005**	$F_{(2,61)} = 9.81$, **p = 0.0002**	$F_{(2,61)} = 5.31$, **p = 0.008**	$t_{(61)} = 3.96$, **p = 0.0006**	$t_{(61)} = 0.68$, p = 0.78	$t_{(61)} = 4.67$, **p = 0.0001**	$t_{(61)} = 1.69$, p = 0.22	$t_{(61)} = -1.50$, p = 0.30	$t_{(61)} = 0.23$, p = 0.97	$t_{(61)} = 1.58$, p = 0.12	$t_{(61)} = 4.12$, **p = 0.0001**	$t_{(61)} = -0.54$, p = 0.59

Supplementary Table 11. Sholl analysis.

Distance from soma - radius [µm]	I RLN3 - mean ± SD (n)		I pCCK- mean ± SD (n)		II - mean ± SD (n)		Two-way ANOVA (type III) - effects (p and F values with df)			Post hoc Tukey test (p and t values with df)								
										Ctrl			MS			Crtl vs. MS		
	Ctrl (10)	MS (10)	Ctrl (12)	MS (12)	Ctrl (10)	MS (13)	Maternal separation	Type	Interaction	I RLN3 vs. I pCCK	I RLN3 vs. II	I pCCK vs. II	I RLN3 vs. I pCCK	I RLN3 vs. II	I pCCK vs. II	I RLN3	I pCCK	II
10	3.1 ± 1.7	2.9 ± 1.1	3.6 ± 1.2	3.0 ± 1.8	2.7 ± 1.2	2.5 ± 1.1	$F_{(1,61)}$ = 0.88, p = 0.35	$F_{(2,61)}$ = 1.41, p = 0.25	$F_{(2,61)}$ = 0.17, p = 0.85	-	-	-	-	-	-	-	-	-
20	4.1 ± 2.0	3.2 ± 0.6	5.8 ± 2.1	4.7 ± 1.4	3.5 ± 2.1	3.7 ± 1.3	$F_{(1,61)}$ = 2.16, p = 0.15	$F_{(2,61)}$ = 7.08, **p = 0.002**	$F_{(2,61)}$ = 0.98, p = 0.38	$t_{(61)}$ = 2.33, p = 0.06	$t_{(61)}$ = 0.81, p = 0.70	$t_{(61)}$ = 3.18, **p = 0.007**	$t_{(61)}$ = 2.07, p = 0.11	$t_{(61)}$ = -0.71, p = 0.76	$t_{(61)}$ = 1.47, p = 0.31	$t_{(61)}$ = 1.22, p = 0.23	$t_{(61)}$ = 1.61, p = 0.11	$t_{(61)}$ = 0.28, p = 0.78
30	4.4 ± 2.1	4.2 ± 1.8	6.2 ± 3.0	5.3 ± 2.3	3.4 ± 1.7	3.8 ± 1.6	$F_{(1,61)}$ = 0.18, p = 0.67	$F_{(2,61)}$ = 5.75, **p = 0.005**	$F_{(2,61)}$ = 0.59, p = 0.56	$t_{(61)}$ = 1.93, p = 0.14	$t_{(61)}$ = 1.04, p = 0.55	$t_{(61)}$ = 3.02, **p = 0.01**	$t_{(61)}$ = 1.15, p = 0.49	$t_{(61)}$ = 0.39, p = 0.92	$t_{(61)}$ = 1.64, p = 0.24	$t_{(61)}$ = 1.04, p = 0.30	$t_{(61)}$ = -0.49, p = 0.62	$t_{(61)}$ = 0.21, p = 0.84
40	4.8 ± 1.9	3.6 ± 0.7	6.1 ± 1.4	4.5 ± 1.4	3.7 ± 2.3	4.1 ± 1.8	$F_{(1,61)}$ = 3.52, p = 0.07	$F_{(2,61)}$ = 4.55, **p = 0.01**	$F_{(2,61)}$ = 1.63, p = 0.21	$t_{(61)}$ = 1.82, p = 0.17	$t_{(61)}$ = 1.50, p = 0.30	$t_{(61)}$ = 3.39, **p = 0.004**	$t_{(61)}$ = 1.28, p = 0.41	$t_{(61)}$ = -0.69, p = 0.77	$t_{(61)}$ = 0.64, p = 0.80	$t_{(61)}$ = 1.63, p = 0.11	$t_{(61)}$ = 2.36, **p = 0.02**	$t_{(61)}$ = -0.55, p = 0.59
50	5.0 ± 1.4	3.6 ± 1.0	6.2 ± 1.9	4.8 ± 1.8	4.5 ± 2.5	4.4 ± 2.3	$F_{(1,61)}$ = 4.39, **p = 0.04**	$F_{(2,61)}$ = 2.53, p = 0.09	$F_{(2,61)}$ = 0.87, p = 0.43	$t_{(61)}$ = 1.44, p = 0.33	$t_{(61)}$ = 0.59, p = 0.83	$t_{(61)}$ = 2.05, p = 0.11	$t_{(61)}$ = 1.42, p = 0.34	$t_{(61)}$ = -0.98, p = 0.59	$t_{(61)}$ = 0.48, p = 0.88	$t_{(61)}$ = 1.65, p = 0.10	$t_{(61)}$ = 1.83, p = 0.07	$t_{(61)}$ = 0.14, p = 0.89
60	4.9 ± 1.7	3.5 ± 0.8	7.0 ± 1.9	4.0 ± 1.6	3.8 ± 2.1	4.3 ± 2.1	$F_{(1,61)}$ = 8.96, **p = 0.004**	$F_{(2,61)}$ = 4.72, **p = 0.01**	$F_{(2,61)}$ = 5.76, **p = 0.005**	$t_{(61)}$ = 2.78, **p = 0.02**	$t_{(61)}$ = 1.40, p = 0.35	$t_{(61)}$ = 4.24, **p = 0.0002**	$t_{(61)}$ = 0.66, p = 0.79	$t_{(61)}$ = -1.10, p = 0.52	$t_{(61)}$ = -0.44, p = 0.91	$t_{(61)}$ = 1.76, p = 0.08	$t_{(61)}$ = 4.17, **p = 0.0001**	$t_{(61)}$ = -0.68, p = 0.50
70	4.1 ± 2.0	3.4 ± 1.1	6.8 ± 2.1	3.7 ± 1.7	4.3 ± 2.2	4.2 ± 2.0	$F_{(1,61)}$ = 7.88, **p = 0.007**	$F_{(2,61)}$ = 3.43, **p = 0.04**	$F_{(2,61)}$ = 3.93, **p = 0.03**	$t_{(61)}$ = 3.26, **p = 0.005**	$t_{(61)}$ = -0.24, p = 0.97	$t_{(61)}$ = 3.01, **p = 0.01**	$t_{(61)}$ = 0.33, p = 0.94	$t_{(61)}$ = -0.94, p = 0.62	$t_{(61)}$ = -0.64, p = 0.80	$t_{(61)}$ = 0.82, p = 0.41	$t_{(61)}$ = 3.98, **p = 0.0002**	$t_{(61)}$ = 0.18, p = 0.86
80	4.1 ± 2.5	3.6 ± 1.2	6.3 ± 2.3	3.4 ± 1.9	4.3 ± 2.3	4.2 ± 2.1	$F_{(1,61)}$ = 5.30, **p = 0.03**	$F_{(2,61)}$ = 1.36, p = 0.27	$F_{(2,61)}$ = 2.99, p = 0.06	$t_{(61)}$ = 2.49, **p = 0.04**	$t_{(61)}$ = -0.21, p = 0.98	$t_{(61)}$ = 2.26, p = 0.07	$t_{(61)}$ = 0.20, p = 0.98	$t_{(61)}$ = -0.63, p = 0.81	$t_{(61)}$ = -0.88, p = 0.66	$t_{(61)}$ = 0.53, p = 0.60	$t_{(61)}$ = 3.40, **p = 0.001**	$t_{(61)}$ = 0.17, p = 0.87
90	3.4 ± 2.1	3.0 ± 1.2	6.0 ± 2.1	3.0 ± 1.8	3.8 ± 2.0	3.9 ± 2.1	$F_{(1,61)}$ = 5.41, **p = 0.02**	$F_{(2,61)}$ = 2.53, p = 0.09	$F_{(2,61)}$ = 4.45, **p = 0.02**	$t_{(61)}$ = 3.18, **p = 0.007**	$t_{(61)}$ = -0.47, p = 0.89	$t_{(61)}$ = 2.69, **p = 0.03**	$t_{(61)}$ = 0.00, p = 1.00	$t_{(61)}$ = -1.15, p = 0.49	$t_{(61)}$ = -1.21, p = 0.45	$t_{(61)}$ = 0.47, p = 0.64	$t_{(61)}$ = 3.84, **p = 0.0003**	$t_{(61)}$ = -0.15, p = 0.89
100	3.3 ± 2.3	2.6 ± 1.3	5.0 ± 1.6	2.8 ± 1.7	3.8 ± 2.0	3.8 ± 1.6	$F_{(1,61)}$ = 4.62, **p = 0.04**	$F_{(2,61)}$ = 1.89, p = 0.10	$F_{(2,61)}$ = 4.45, p = 0.16	$t_{(61)}$ = 2.23, p = 0.07	$t_{(61)}$ = -0.63, p = 0.81	$t_{(61)}$ = 1.57, p = 0.26	$t_{(61)}$ = 0.31, p = 0.95	$t_{(61)}$ = -1.66, p = 0.23	$t_{(61)}$ = -1.42, p = 0.34	$t_{(61)}$ = 0.88, p = 0.38	$t_{(61)}$ = 2.98, **p = 0.004**	$t_{(61)}$ = -0.06, p = 0.95
110	3.2 ± 2.1	2.2 ± 1.4	4.8 ± 1.9	2.7 ± 1.4	3.7 ± 1.7	3.5 ± 1.5	$F_{(1,61)}$ = 7.19, **p = 0.009**	$F_{(2,61)}$ = 2.44, p = 0.10	$F_{(2,61)}$ = 2.09, p = 0.13	$t_{(61)}$ = 2.27, p = 0.07	$t_{(61)}$ = -0.66, p = 0.79	$t_{(61)}$ = 1.57, p = 0.27	$t_{(61)}$ = 0.65, p = 0.80	$t_{(61)}$ = -1.89, p = 0.15	$t_{(61)}$ = -1.29, p = 0.40	$t_{(61)}$ = 1.33, p = 0.19	$t_{(61)}$ = 3.15, **p = 0.003**	$t_{(61)}$ = 0.23, p = 0.82
120	3.1 ± 2.2	2.1 ± 1.4	4.1 ± 1.6	2.6 ± 1.4	3.0 ± 0.9	3.0 ± 1.5	$F_{(1,61)}$ = 4.82, **p = 0.03**	$F_{(2,61)}$ = 1.23, p = 0.30	$F_{(2,61)}$ = 1.41, p = 0.25	$t_{(61)}$ = 1.49, p = 0.30	$t_{(61)}$ = 0.15, p = 0.99	$t_{(61)}$ = 1.57, p = 0.28	$t_{(61)}$ = 0.73, p = 0.75	$t_{(61)}$ = -1.39, p = 0.36	$t_{(61)}$ = 0.67, p = 0.78	$t_{(61)}$ = 1.45, p = 0.15	$t_{(61)}$ = 2.38, **p = 0.02**	$t_{(61)}$ = 0.00, p = 1.00
130	2.8 ± 1.9	1.7 ± 1.3	3.5 ± 1.6	2.3 ± 1.6	3.1 ± 1.0	2.5 ± 1.6	$F_{(1,61)}$ = 6.43, **p = 0.01**	$F_{(2,61)}$ = 0.20, p = 0.31	$F_{(2,61)}$ = 0.27, p = 0.76	$t_{(61)}$ = 1.08, p = 0.53	$t_{(61)}$ = -0.44, p = 0.90	$t_{(61)}$ = 0.62, p = 0.81	$t_{(61)}$ = 0.98, p = 0.59	$t_{(61)}$ = -1.32, p = 0.39	$t_{(61)}$ = 0.34, p = 0.94	$t_{(61)}$ = 1.63, p = 0.11	$t_{(61)}$ = 1.89, p = 0.06	$t_{(61)}$ = 0.88, p = 0.38
140	2.5 ± 1.4	1.6 ± 1.2	3.2 ± 1.5	2.3 ± 1.5	2.9 ± 1.0	2.1 ± 1.5	$F_{(1,61)}$ = 6.82, **p = 0.01**	$F_{(2,61)}$ = 1.28, p = 0.28	$F_{(2,61)}$ = 0.01, p = 0.99	$t_{(61)}$ = 1.14, p = 0.50	$t_{(61)}$ = -0.65, p = 0.79	$t_{(61)}$ = 0.45, p = 0.89	$t_{(61)}$ = 1.11, p = 0.51	$t_{(61)}$ = -0.83, p = 0.69	$t_{(61)}$ = 0.32, p = 0.95	$t_{(61)}$ = 1.47, p = 0.15	$t_{(61)}$ = 1.64, p = 0.11	$t_{(61)}$ = 1.43, p = 0.16
150	2.0 ± 1.1	1.5 ± 1.1	2.9 ± 1.5	2.2 ± 1.5	2.6 ± 1.1	2.0 ± 1.4	$F_{(1,61)}$ = 3.71, p = 0.06	$F_{(2,61)}$ = 2.08, p = 0.13	$F_{(2,61)}$ = 0.05, p = 0.95	-	-	-	-	-	-	-	-	-
160	1.7 ± 0.9	1.4 ± 1.0	3.0 ± 1.7	1.9 ± 1.6	2.4 ± 1.2	1.8 ± 1.4	$F_{(1,61)}$ = 4.13, **p = 0.046**	$F_{(2,61)}$ = 2.50, p = 0.09	$F_{(2,61)}$ = 0.47, p = 0.63	$t_{(61)}$ = 2.26, p = 0.07	$t_{(61)}$ = -1.17, p = 0.48	$t_{(61)}$ = 1.04, p = 0.55	$t_{(61)}$ = 0.90, p = 0.64	$t_{(61)}$ = -0.65, p = 0.79	$t_{(61)}$ = 0.27, p = 0.96	$t_{(61)}$ = 0.50, p = 0.62	$t_{(61)}$ = 1.98, p = 0.05	$t_{(61)}$ = 1.12, p = 0.27
170	1.6 ± 0.8	1.4 ± 1.0	2.6 ± 2.2	1.8 ± 1.5	2.3 ± 1.3	2.0 ± 1.8	$F_{(1,61)}$ = 1.19, p = 0.28	$F_{(2,61)}$ = 1.35, p = 0.27	$F_{(2,61)}$ = 0.20, p = 0.82	-	-	-	-	-	-	-	-	-
180	1.4 ± 1.0	1.5 ± 1.1	2.1 ± 1.7	1.6 ± 1.5	2.4 ± 1.5	1.8 ± 1.5	$F_{(1,61)}$ = 0.83, p = 0.37	$F_{(2,61)}$ = 1.20, p = 0.31	$F_{(2,61)}$ = 0.34, p = 0.71	-	-	-	-	-	-	-	-	-
190	1.4 ± 1.0	1.1 ± 1.0	1.8 ± 1.9	1.4 ± 1.2	2.2 ± 1.4	1.8 ± 1.5	$F_{(1,61)}$ = 1.28, p = 0.26	$F_{(2,61)}$ = 1.51, p = 0.23	$F_{(2,61)}$ = 0.01, p = 0.99	-	-	-	-	-	-	-	-	-
200	1.4 ± 1.0	1.1 ± 1.0	2.0 ± 2.4	1.3 ± 1.1	2.1 ± 1.4	1.8 ± 1.6	$F_{(1,61)}$ = 1.32, p = 0.25	$F_{(2,61)}$ = 1.17, p = 0.32	$F_{(2,61)}$ = 0.18, p = 0.83	-	-	-	-	-	-	-	-	-
210	1.2 ± 1.0	1.1 ± 1.0	1.5 ± 1.4	1.1 ± 1.0	2.0 ± 1.4	1.7 ± 1.4	$F_{(1,61)}$ = 0.84, p = 0.36	$F_{(2,61)}$ = 1.99, p = 0.15	$F_{(2,61)}$ = 0.09, p = 0.91	-	-	-	-	-	-	-	-	-
220	1.1 ± 1.0	1.1 ± 1.0	1.3 ± 1.3	0.8 ± 0.8	1.7 ± 1.1	1.5 ± 1.1	$F_{(1,61)}$ = 1.11, p = 0.30	$F_{(2,61)}$ = 1.76, p = 0.18	$F_{(2,61)}$ = 0.43, p = 0.66	-	-	-	-	-	-	-	-	-
230	0.9 ± 1.0	1.0 ± 0.9	1.3 ± 1.3	0.7 ± 0.7	1.8 ± 1.3	1.1 ± 1.0	$F_{(1,61)}$ = 2.80, p = 0.10	$F_{(2,61)}$ = 1.46, p = 0.24	$F_{(2,61)}$ = 1.01, p = 0.37	-	-	-	-	-	-	-	-	-
240	0.8 ± 0.9	0.9 ± 1.0	0.9 ± 0.5	0.6 ± 0.7	1.6 ± 0.8	1.2 ± 1.1	$F_{(1,61)}$ = 1.17, p = 0.28	$F_{(2,61)}$ = 3.54, **p = 0.04**	$F_{(2,61)}$ = 1.01, p = 0.38	$t_{(61)}$ = 0.32, p = 0.95	$t_{(61)}$ = -2.10, p = 0.10	$t_{(61)}$ = -1.87, p = 0.16	$t_{(61)}$ = -0.87, p = 0.66	$t_{(61)}$ = -0.71, p = 0.76	$t_{(61)}$ = -1.67, p = 0.23	$t_{(61)}$ = -0.26, p = 0.79	$t_{(61)}$ = 0.96, p = 0.34	$t_{(61)}$ = 1.24, p = 0.22
250	0.7 ± 0.7	0.8 ± 0.9	0.8 ± 0.6	0.6 ± 0.7	1.6 ± 1.5	0.8 ± 0.9	$F_{(1,61)}$ = 1.82, p = 0.18	$F_{(2,61)}$ = 2.23, p = 0.12	$F_{(2,61)}$ = 1.20, p = 0.31	-	-	-	-	-	-	-	-	-
260	0.7 ± 0.7	0.7 ± 0.7	0.8 ± 0.5	0.6 ± 0.7	1.3 ± 1.2	0.5 ± 0.7	$F_{(1,61)}$ = 3.37, p = 0.07	$F_{(2,61)}$ = 0.56, p = 0.57	$F_{(2,61)}$ = 2.02, p = 0.14	-	-	-	-	-	-	-	-	-
270	0.7 ± 0.7	0.4 ± 0.5	0.8 ± 0.5	0.5 ± 0.7	1.2 ± 1.1	0.5 ± 0.7	$F_{(1,61)}$ = 6.09, **p = 0.02**	$F_{(2,61)}$ = 0.92, p = 0.41	$F_{(2,61)}$ = 0.82, p = 0.45	$t_{(61)}$ = 0.17, p = 0.99	$t_{(61)}$ = -1.58, p = 0.26	$t_{(61)}$ = -1.48, p = 0.31	$t_{(61)}$ = 0.33, p = 0.94	$t_{(61)}$ = -0.21, p = 0.98	$t_{(61)}$ = 0.14, p = 0.99	$t_{(61)}$ = 0.95, p = 0.35	$t_{(61)}$ = 0.87, p = 0.39	$t_{(61)}$ = 2.48, **p = 0.01**
280	0.7 ± 0.7	0.4 ± 0.5	0.8 ± 0.5	0.5 ± 0.7	0.9 ± 1.2	0.5 ± 0.7	$F_{(1,61)}$ = 3.44, p = 0.07	$F_{(2,61)}$ = 0.17, p = 0.84	$F_{(2,61)}$ = 0.82, p = 0.47	-	-	-	-	-	-	-	-	-
290	0.6 ± 0.5	0.3 ± 0.5	0.8 ± 0.5	0.4 ± 0.5	0.8 ± 1.0	0.5 ± 0.7	$F_{(1,61)}$ = 4.33, **p = 0.04**	$F_{(2,61)}$ = 0.46, p = 0.63	$F_{(2,61)}$ = 0.01, p = 0.99	$t_{(61)}$ = 0.55, p = 0.85	$t_{(61)}$ = -0.71, p = 0.76	$t_{(61)}$ = -0.18, p = 0.98	$t_{(61)}$ = 0.43, p = 0.90	$t_{(61)}$ = -0.61, p = 0.82	$t_{(61)}$ = -0.18, p = 0.98	$t_{(61)}$ = 1.06, p = 0.29	$t_{(61)}$ = 1.29, p = 0.20	$t_{(61)}$ = 1.27, p = 0.21
300	0.6 ± 0.5	0.3 ± 0.5	0.7 ± 0.5	0.6 ± 0.9	0.8 ± 1.0	0.4 ± 0.7	$F_{(1,61)}$ = 2.33, p = 0.13	$F_{(2,61)}$ = 0.36, p = 0.70	$F_{(2,61)}$ = 0.33, p = 0.72	-	-	-	-	-	-	-	-	-
310	0.6 ± 0.5	0.3 ± 0.5	0.6 ± 0.5	0.4 ± 0.7	0.7 ± 0.9	0.4 ± 0.7	$F_{(1,61)}$ = 2.69, p = 0.11	$F_{(2,61)}$ = 0.11, p = 0.90	$F_{(2,61)}$ = 0.09, p = 0.91	-	-	-	-	-	-	-	-	-
320	0.6 ± 0.5	0.3 ± 0.5	0.6 ± 0.7	0.4 ± 0.7	0.7 ± 0.9	0.5 ± 0.8	$F_{(1,61)}$ = 1.88, p = 0.18	$F_{(2,61)}$ = 0.19, p = 0.83	$F_{(2,61)}$ = 0.05, p = 0.95	-	-	-	-	-	-	-	-	-

The symbol (-) indicates no post-hoc analysis

Supplementary Table 12. Measured morphological parameters - descriptive statistics.

Morphological parameters	I RLN3 - mean ± SD (n)		I pCCK- mean ± SD (n)		II - mean ± SD (n)	
	Ctrl (10)	MS (10)	Ctrl (12)	MS (12)	Ctrl (10)	MS (13)
Nb_prim	3.3 ± 1.2	3.0 ± 0.8	3.8 ± 1.0	3.9 ± 0.8	3.0 ± 0.8	3.0 ± 0.7
Nb_bif	5.5 ± 3.3	3.5 ± 2.0	8.2 ± 2.9	4.8 ± 1.7	6.7 ± 3.0	6.3 ± 5.3
Nb_branch	14.0 ± 6.4	9.6 ± 3.6	19.9 ± 5.6	13.2 ± 3.4	16.3 ± 6.5	15.2 ± 10.6
Nb_tips	8.5 ± 3.2	6.1 ± 1.7	11.8 ± 2.9	8.3 ± 1.8	9.6 ± 3.6	8.9 ± 5.4
Max_branch	2.7 ± 0.8	2.0 ± 1.1	3.7 ± 1.1	2.7 ± 1.2	3.3 ± 1.3	3.0 ± 1.6
Tot_dendr [µm]	970.7 ± 334.4	765.6 ± 313.3	1402.0 ± 422.5	912.0 ± 449.8	1081.0 ± 392.4	992.5 ± 488.1

Abbreviations: Max_branch, Maximal branch order; Nb_bif, Number of bifurcations; Nb_branch, Number of branches; Nb_prim, Number of primary dendrites; Nb_tips, Number of dendritic tips; Tot_dendr, Total dendritic length

Supplementary Table 13. Correlation matrix of the measured morphological parameters.

	Nb_prim	Nb_bif	Nb_branch	Nb_tips	Max_branch
**Nb_bif**	-0.01				
**Nb_branch**	0.1	0.99*			
**Nb_tips**	0.21	0.96*	0.99*		
**Max_branch**	-0.03	0.84*	0.83*	0.81*	
**Tot_dendr**	0.07	0.70*	0.71*	0.70*	0.62*

* - Correlation significant at level $p \leq 0.05$

Abbreviations: Max_branch, Maximal branch order; Nb_bif, Number of bifurcations; Nb_branch, Number of branches; Nb_prim, Number of primary dendrites; Nb_tips, Number of dendritic tips; Tot_dendr, Total dendritic length

Supplementary Table 14. Correlation matrix of principal components and measured morphological parameters.

Correlation matrix	PC1	PC2
Nb_prim	0.10	-0.99*
Nb_bif	0.98*	0.10
Nb_branch	0.98*	-0.01
Nb_tips	0.97*	-0.12
Max_branch	0.89*	0.14
Tot_dendr	0.80*	0.00

Abbreviations: Max_branch, Maximal branch order; Nb_bif, Number of bifurcations; Nb_branch, Number of branches; Nb_prim, Number of primary dendrites; Nb_tips, Number of dendritic tips; Tot_dendr, Total dendritic length

Supplementary Table 15. Loadings of principal components.

Loadings	PC1	PC2
Nb_prim	0.05	-0.98
Nb_bif	0.47	0.10
Nb_branch	0.48	-0.01
Nb_tips	0.47	-0.12
Max_branch	0.43	0.14
Tot_dendr	0.38	0.002

Abbreviations: Max_branch, Maximal branch order; Nb_bif, Number of bifurcations; Nb_branch, Number of branches; Nb_prim, Number of primary dendrites; Nb_tips, Number of dendritic tips; Tot_dendr, Total dendritic length

Supplementary Table 16. Statistical analysis of principal components.

	Two-way ANOVA - effects (F and p values)			Post hoc Tukey test (t and p values)		
	Maternal separation	Type	Interaction	C I RLN3 vs. MS I RLN3	C I pCCK vs. MS I pCCK	C II vs. MS II
PC1	$F_{(1, 61)} = 7.27$ **p = 0.009**	$F_{(2, 61)} = 3.64$ **p = 0.03**	$F_{(2, 61)} = 1.14$ p = 0.328	$t_{(61)} = 1.52$ p = 0.13	$t_{(61)} = 2.68$ **p = 0.009**	$t_{(61)} = 0.50$ p = 0.62
PC2	$F_{(1, 61)} = 0.0006$ p = 0.98	$F_{(2, 61)} = 5.44$ **p = 0.007**	$F_{(2, 61)} = 0.40$ p = 0.67	$t_{(61)} = -0.61$ p = 0.54	$t_{(61)} = 0.67$ p = 0.51	$t_{(61)} = 0.05$ p = 0.96
	Post hoc Tukey test (p and t values) - continued					
	C I RLN3 vs. C I pCCK	C I RLN3 vs. C II	C I pCCK vs. C II	MS I RLN3 vs. MS I pCCK	MS I RLN3 vs. MS II	MS I pCCK vs. MS II
PC1	$t_{(61)} = 2.32$ p = 0.06	$t_{(61)} = -0.87$ p = 0.66	$t_{(61)} = 1.41$ p = 0.34	$t_{(61)} = 1.35$ p = 0.37	$t_{(61)} = -2.04$ p = 0.11	$t_{(61)} = -0.70$ p = 0.77
PC2	$t_{(61)} = -0.97$ p = 0.6	$t_{(61)} = -0.87$ p = 0.66	$t_{(61)} = -1.88$ p = 0.15	$t_{(61)} = -2.24$ p = 0.07	$t_{(61)} = -2.64$ **p = 0.03**	$t_{(61)} = -0.23$ p = 0.97

Supplementary Table 17. mRNA expression: numbers of all counted cells.

NI part	Mean ± SD (n)		p and t values with df	test
	Ctrl (5)	MS (6)		
NI	125 ± 20	126 ± 12	$t_{(9)} = 0.07$, p = 0.94	t-test
NIc	72 ± 13	75 ± 11	$t_{(9)} = 0.51$, p = 0.62	t-test
NId	54 ± 7	51 ± 9	$t_{(9)} = 0.61$, p = 0.56	t-test

Supplementary Table 18. Different mRNA species expression in the NI of MS and control rats.

mRNA species	Mean ± SD (% of counted cells/rat) (n)				Two-way ANOVA - effects (p and F values with df)		
	Ctrl (5)		MS (6)		Maternal separation	Localization	Interaction
	NIc	NId	NIc	NId			
vGAT1+	84 ± 5	41 ± 8	83 ± 11	49 ± 8	$F_{(1,18)} = 1.004$, $p = 0.33$	$F_{(1,18)} = 177.7$, **$p < 0.0001$**	$F_{(1,18)} = 1.76$, $p = 0.20$
vGlut2+	19 ± 4	64 ± 10	19 ± 8	56 ± 9	$F_{(1,18)} = 1.64$, $p = 0.22$	$F_{(1,18)} = 142.1$, **$p < 0.0001$**	$F_{(1,18)} = 1.55$, $p = 0.23$
Rln3+	47 ± 6	18 ± 4	46 ± 8	24 ± 4	$F_{(1,18)} = 0.82$, $p = 0.38$	$F_{(1,18)} = 101.5$, **$p < 0.0001$**	$F_{(1,18)} = 1.6$, $p = 0.22$
Cck+	32 ± 8	3 ± 3	37 ± 8	3 ± 2	$F_{(1,18)} = 1.25$, $p = 0.28$	$F_{(1,18)} = 154.4$, **$p < 0.0001$**	$F_{(1,18)} = 0.67$, $p = 0.42$
CRHR1+	72 ± 9	40 ± 11	81 ± 6	55 ± 9	$F_{(1,18)} = 9.65$, **$p = 0.006$**	$F_{(1,18)} = 59.5$, **$p < 0.0001$**	$F_{(1,18)} = 0.41$, $p = 0.53$
TrkA+	15 ± 3	18 ± 9	26 ± 9	29 ± 16	$F_{(1,18)} = 5.31$, **$p = 0.03$**	$F_{(1,18)} = 0.5$, $p = 0.49$	$F_{(1,18)} = 8E-07$, $p = 0.999$

Supplementary Table 19. CRHR1 mRNA-expressing cells.

mRNA combination	Mean ± SD (% of counted cells/rat) (n)		p and t values with df	test
	Ctrl (5)	MS (6)		
**All CRHR1**				
Nl	58 ± 8	71 ± 5	$t_{(9)}$ = 3.14, **p = 0.01**	t-test
Nlc	69 ± 4 (4)	83 ± 12	U = 0, **p = 0.0095**	M-W
Nld	40 ± 11	54 ± 9	$t_{(9)}$ = 2.31, **p = 0.047**	t-test
CRHR1 + vGAT1				
Nl	48 ± 7	57 ± 5	$t_{(9)}$ = 2.2, p = 0.056	t-test
Nlc	66 ± 10	73 ± 9	$t_{(9)}$ = 1.16, p = 0.27	t-test
Nld	24 ± 6	32 ± 4	$t_{(9)}$ = 2.61, **p = 0.03**	t-test
CRHR1 + vGlut2				
Nl	13 ± 5	16 ± 9	$t_{(9)}$ = 0.7, p = 0.5	t-test
Nlc	8 ± 2	10 ± 8	$t_{(5.88)}$ = 0.51, p = 0.63	Welch
Nld	20 ± 10	26 ± 11	$t_{(9)}$ = 0.93, p = 0.38	t-test
CRHR1 + Rln3				
Nl	33 ± 4	36 ± 6	$t_{(9)}$ = 1.31, p = 0.29	t-test
Nlc	41 ± 1 (4)	45 ± 8	$t_{(5.29)}$ = 1.04, p = 0.34	Welch
Nld	18 ± 4	23 ± 4	$t_{(9)}$ = 2.05, p = 0.07	t-test
CRHR1+ Cck				
Nl	16 ± 7	21 ± 5	$t_{(9)}$ = 1.61, p = 0.14	t-test
Nlc	26 ± 10	34 ± 9	$t_{(9)}$ = 1.39, p = 0.2	t-test
Nld	2 ± 2	3 ± 1	$t_{(9)}$ = 1.23, p = 0.25	t-test

Supplementary Table 20. Mean area fraction of CRHR1 and TrkA-immunofluorescent dots per single cell.

mRNA species	Mean ± SD (area fraction of counted cells/rat x10^{-3})				Two-way ANOVA - effects (p and F values with df)		
	Ctrl (n)		MS (n)		Maternal separation	Localization	Interaction
	Nlc (5)	Nld (5)	Nlc (6)	Nld (5)			
CRHR1+	2.46 ± 1.06	2.53 ± 1.06	2.97 ± 0.74	3.74 ± 0.63	$F_{(1,17)}$ = 5.04, **p = 0.04**	$F_{(1,17)}$ = 1.22, p = 0.29	$F_{(1,17)}$ = 1.22, p = 0.40
TrkA+	0.89 ± 0.34	2.02 ± 0.88	1.28 ± 0.71	3.76 ± 1.67	$F_{(1,17)}$ = 5.89, **p = 0.03**	$F_{(1,17)}$ = 16.80, **p = 0.0008**	$F_{(1,17)}$ = 2.34, p = 0.14

Supplementary Table 21. TrkA mRNA-expressing cells.

mRNA combination	Mean ± SD/ Median ± iqr (% of counted cells/rat) (n)		p and t values with df	test
	Ctrl (5)	MS (6)		
**All TrkA**				
NI	14 ± 2 (4)	27 ± 12	$t_{(5.35)} = 2.66$, **p = 0.04**	Welch
NIc	14 ± 1 (4)	25 ± 13	$t_{(5.03)} = 3.35$, **p = 0.02**	Welch
NId	18 ± 9	29 ± 16	$t_{(9)} = 1.3$, p = 0.23	t-test
TrkA + vGAT				
NI	21 ± 3 (4)	33 ± 22	U = 0, **p = 0.0095**	M-W
NIc	13 ± 1 (4)	23 ± 7	$t_{(5.35)} = 3.53$, **p = 0.02**	Welch
NId	9 ± 4	15 ± 9	$t_{(9)} = 1.61$, p = 0.14	t-test
TrkA + vGlut2				
NI	10 ± 2 (4)	20 ± 13	$t_{(5.54)} = 1.88$, p = 0.11	Welch
NIc	1 ± 2	4 ± 3	$t_{(9)} = 1.92$, p = 0.09	t-test
NId	9 ± 2 (4)	15 ± 10	$t_{(5.69)} = 1.6$, p = 0.16	Welch
TrkA + Rln3				
NI	6 ± 4	12 ± 4	$t_{(9)} = 2.74$, **p = 0.02**	t-test
NIc	8 ± 4	14 ± 3	$t_{(9)} = 2.77$, **p = 0.02**	t-test
NId	4 ± 3	9 ± 6	$t_{(9)} = 1.77$, p = 0.11	t-test
TrkA + Cck				
NI	3 ± 1	6 ± 2	$t_{(9)} = 2.11$, p = 0.06	t-test
NIc	4 ± 1 (4)	9 ± 4	$t_{(5.32)} = 2.91$, **p = 0.03**	Welch
NId	1 ± 1	1 ± 1	$t_{(9)} = 0.53$, p = 0.61	t-test
TrkA + CRHR1				
NI	10 ± 4	18 ± 7	$t_{(9)} = 2.53$, **p = 0.03**	t-test
NIc	13 ± 4	22 ± 6	$t_{(9)} = 2.83$, **p = 0.02**	t-test
NId	6 ± 3	13 ± 9	$t_{(9)} = 1.69$, p = 0.13	t-test

Supplementary Table 22. All RLN3 mRNA-expressing cells.

mRNA Combination	Mean ± SD (% of counted cells/rat) (n)		p and t values with df	test
	Ctrl (5)	MS (6)		
All Rln3+				
NI	34 ± 4	37 ± 6	$t_{(9)} = 0.86$, $p = 0.41$	t-test
NIc	47 ± 6	46 ± 8	$t_{(9)} = 0.20$, $p = 0.84$	t-test
NId	18 ± 4	24 ± 4	$t_{(9)} = 2.25$, **$p = 0.05$**	t-test
